# Letermovir for cytomegalovirus prophylaxis after allogeneic hematopoietic stem cell transplantation in acute leukemia patients: results of the LETreal study

**DOI:** 10.3389/fimmu.2026.1923003

**Published:** 2026-09-03

**Authors:** Yi Xia, Qi Wen, Jing Liu, Jinfang Zhao, Xiaohui Zhang, Lanping Xu, Yu Wang, Yuhong Chen, Yuanyuan Zhang, Yuqian Sun, Yifei Cheng, Yao Chen, Wei Han, Fengrong Wang, Jingzhi Wang, Jun Kong, Leqing Cao, Daoxing Deng, Xiaojun Huang, Xiaodong Mo

**Affiliations:** 1 Peking University People’s Hospital, Peking University Institute of Hematology, National Clinical Research Center for Hematologic Disease, Beijing Key Laboratory of Cell and Gene Therapy for Hematologic Malignancies, Peking University, Beijing, China; 2 Peking-Tsinghua Center for Life Sciences, Academy for Advanced Interdisciplinary Studies, Peking University, Beijing, China

**Keywords:** acute leukemia, allogeneic hematopoietic stem cell transplantation, cytomegalovirus, letermovir, prophylaxis

## Abstract

**Objective:**

Cytomegalovirus (CMV) infection can cause severe morbidity and mortality in patients undergoing allogeneic hematopoietic stem cell transplantation (allo-HSCT). Letermovir is recommended as CMV prophylaxis for allo-HSCT recipients. This study aimed to further confirm the efficacy of letermovir prophylaxis in different subgroups based on a large cohort of acute leukemia patients following allo-HSCT.

**Methods:**

1331 acute leukemia patients who underwent allo-HSCT (including 978 and 353 patients with and without letermovir prophylaxis, respectively) were enrolled. The occurrence of clinically significant CMV infection (cs-CMVi), CMV disease, and refractory CMV infection were evaluated. Propensity score matching (PSM) was applied to balance baseline characteristics between patients with and without letermovir.

**Results:**

In patients receiving letermovir, the 100-day cumulative incidence of cs-CMVi after allo-HSCT was 7.1% (95% CI, 5.5−8.7%), and this rate was greater in children; however, no variables increased the risk of cs-CMVi at 100 days according to multivariate analysis. The cumulative incidence rates of cs-CMVi and CMV disease between 100 and 200 days after allo-HSCT for patients receiving letermovir prophylaxis were 12.5% (95% CI, 10.3−14.7%) and 1.6% (95% CI, 0.8−2.4%), respectively. For the subgroup of patients who did not experience a CMV infection within the first 100 days, these rates were 11.7% (95% CI, 9.5−13.9%) and 1.4% (95% CI, 0.6−2.2%), respectively. Donor-/recipient+ CMV serostatus (44.2%, HR, 5.00; P<0.001) independently increased the risk of cs-CMVi, and age > 60 years (5.0%, HR, 4.01; P = 0.030) increased the risk of CMV disease between 100 and 200 days after allo-HSCT. After PSM adjustment, letermovir prophylaxis was associated with a lower incidence of cs-CMV infection (grade II–IV: 1.9% vs. 10.7%, P = 0.024; grade III–IV: 0% vs. 27.3%, P<0.001) in patients with acute graft-versus-host disease (GVHD). The incidence of cs-CMVi was 18.4% (95% CI, 7.4−29.4%) among chronic GVHD patients receiving letermovir prophylaxis. The incidence of cs-CMVi between 100 and 200 days after allo-HSCT was comparable between patients with prolonged letermovir prophylaxis and those without letermovir prophylaxis.

**Conclusion:**

We confirmed the efficacy of letermovir prophylaxis in different subgroups, but the subgroup who would benefit most from prolonged prophylaxis should be further clarified in the future.

## 1 Introduction

Allogeneic hematopoietic stem cell transplantation (allo-HSCT) is the most effective means for curing acute leukemia (1–4). However, viral infection, particularly cytomegalovirus (CMV) reactivation, can cause severe morbidity and even mortality (5–7). Letermovir, which has been proven to prevent CMV reactivation in randomized controlled trials (8, 9), is recommended for CMV prophylaxis for allo-HSCT recipients (10).

Recently, several real-world studies have confirmed the efficacy of letermovir in CMV prophylaxis (11). However, some questions remain. For example, whether letermovir prophylaxis can decrease the risk of CMV infection in patients with specific risk factors (e.g., donor-/recipient+ CMV serostatus) is controversial (12–14). No studies have compared the efficacy of letermovir prophylaxis between adults and elderly patients (≥ 60 years) or children (< 16 years). Most of these studies enrolled HSCT recipients that were identical sibling donors (ISDs) or matched unrelated donors (MUDs), and whether haploidentical related donors (HID) HSCT recipients can achieve similar effectiveness of letermovir prophylaxis is unclear (15–19). In particular, most HID HSCT recipients receive posttransplantation cyclophosphamide (PTCy)-based graft-versus-host disease (GVHD) prophylaxis (20, 21), and the evidence concerning the addition of letermovir in HID HSCT using antithymocyte globulin (ATG) is insufficient.

In addition, a randomized, phase 3 trial of patients revealed that extending the duration of letermovir prophylaxis to 200 days following allo-HSCT was efficacious at reducing the incidence of late-onset cs-CMV infection (9). In this study, 60% of the donors in the letermovir group were CMV seropositive. However, the seroprevalence of CMV in the Chinese population is very high; that is, CMV seropositivity continued to increase with age (up to 97% among patients > 20 years old) (22). Thus, we wanted to clarify which patient subgroup would benefit most from prolonged letermovir prophylaxis in the real world.

Several studies have reported that letermovir prophylaxis might be associated with Epstein–Barr virus (EBV) infection or EBV-associated posttransplant lymphoproliferative disorder (PTLD) after allo-HSCT (23–26). However, relatively few patients received letermovir prophylaxis in the studies mentioned above. Thus, the impact of letermovir prophylaxis on EBV infection after allo-HSCT should be further confirmed in the real world.

Thus, in the present real-world study, we aimed to further confirm the efficacy of letermovir prophylaxis in different subgroups on the basis of a large allo-HSCT cohort (letermovir prophylaxis in the real-world study, LETreal study), in which most of them received HID HSCT with an ATG-based protocol for GVHD prophylaxis. We also aimed to confirm the association between letermovir prophylaxis and EBV infection after allo-HSCT.

## 2 Materials and methods

### 2.1 Study design

**Figure 1:**
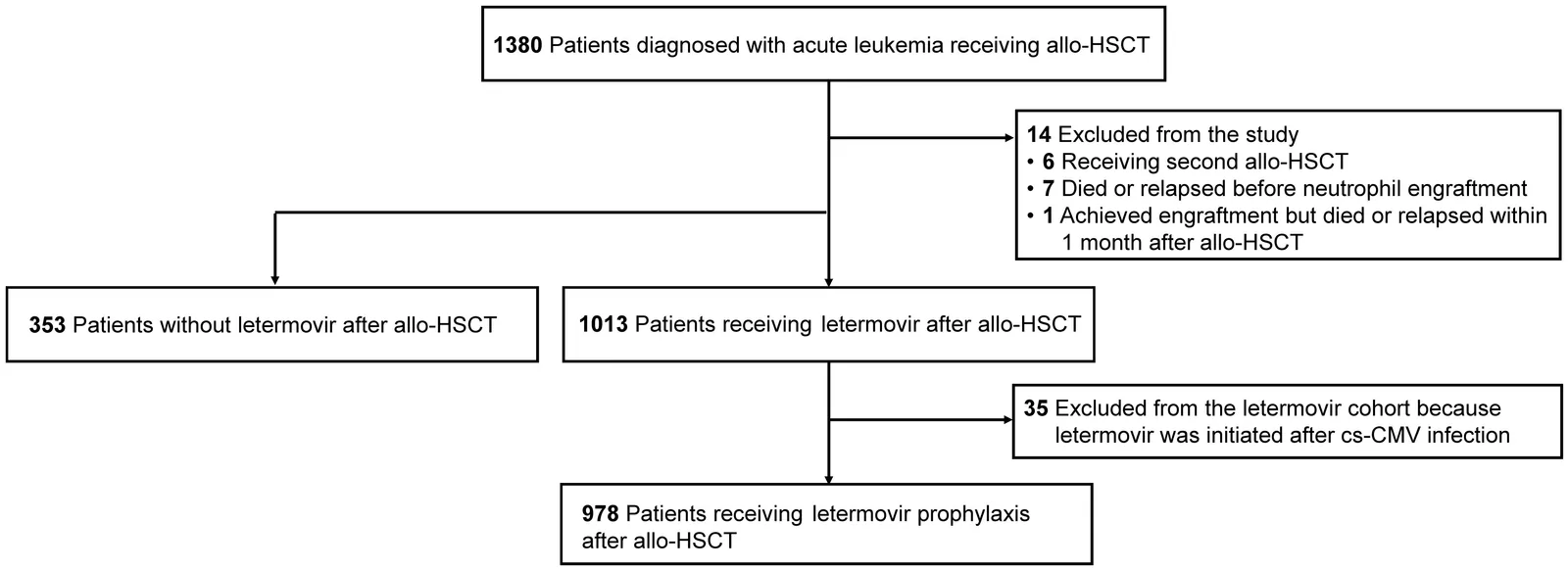
Flow diagram of patient enrollment. Allo-HSCT, allogeneic hematopoietic stem cell transplantation; cs-CMV, clinically significant cytomegalovirus infection.

This retrospective study included consecutive acute leukemia patients who underwent allo-HSCT at the Institute of Hematology, Peking University (PUIH), between January 2, 2023, and March 31, 2025, with the last follow-up conducted on November 30, 2025. Eligible patients for the final analysis were allo-HSCT recipients (regardless of age, donor, and stem-cell source) with acute leukemia who underwent allo-HSCT. The exclusion criteria were as follows: 1) underwent second allo-HSCT; 2) died or relapsed before neutrophil engraftment; 3) achieved engraftment but died or relapsed within 1 month after allo-HSCT; and 4) began letermovir after cs-CMV infection (**Figure 1**). Institutional review board of PUIH approval for this research was obtained (No. 2026PHB231-001), and the study complied with the principles of the *Declaration of Helsinki*.

### 2.2 Transplantation regimens and protocol for infection monitoring and prophylaxis

The major conditioning regimens included cytarabine, busulfan, cyclophosphamide, and semustine (27–29). Granulocyte colony-stimulating factor-primed peripheral blood (PB) harvests were administered to the recipients on the same day of collection. ATG, cyclosporine, mycophenolate mofetil, and short-term methotrexate were administered to prevent GVHD (30). The protocol for acute and chronic GVHD treatment was in accordance with international recommendations (31) and protocols in PUIH (32–35). Detailed information concerning infection prophylaxis and monitoring is provided in the **Supplementary Methods** (36–39).

### 2.3 Definition

CMV DNAemia was defined as the detection of CMV DNA in plasma samples. Clinically significant CMV (cs-CMV) infection was defined as CMV disease (40) or CMV DNAemia leading to preemptive treatment on the basis of CMV DNAemia thresholds at our institute (i.e., > 1×10^3^ copies/mL CMV in a single test on plasma) (8). Refractory CMV infection was defined according to the consensus of the Transplant-Associated Virus Infections Forum (40). CMV activation was treated in accordance with the Chinese Society of Hematology (41).

EBV DNAemia requiring preemptive treatment was defined as cs-EBV infection, which was based on the thresholds at our institute (i.e., > 1×10^3^ copies/mL EBV in a single test on plasma). EBV-PTLD was defined according to international recommendations (42).

### 2.4 Statistical analysis

Comparisons of patient characteristics between the groups were performed using Student’s t test for continuous variables and *χ*^2^ and Fisher’s exact tests for categorical data. The probabilities of survival were calculated using the Kaplan–Meier estimator. Cumulative incidences were estimated for cs-CMV infection, CMV disease, cs-EBV infection, EBV-PTLD, relapse and nonrelapse mortality (NRM) to accommodate competing risks (Fine-Gray model). Hazard ratios (HRs) for CMV and EBV infection were estimated in a multivariate analysis using Cox proportional hazards regression. The effect of GVHD on cs-CMV infection was evaluated using time-dependent Cox proportional hazards models. (6) Independent variables with *P*>0.1 were sequentially excluded from the model, and *P*<0.05 was considered to indicate statistical significance. Propensity score matching (PSM) was applied to balance baseline characteristics using the nearest-neighbor method, including age at allo-HSCT (<16 years vs. 16–60 years vs. > 60 years), sex (male vs. female), donor–recipient relationship (matched sibling donor vs. haploidentical related donor vs. unrelated donor), and GVHD prophylaxis (without ATG vs. ATG), with a 2% caliper. SPSS 26 (SPSS Inc./IBM, Armonk, NY, USA) and the R software package (version 3.6.2; http://www.r-project.org) were used for data analyses.

## 3 Results

### 3.1 Patient characteristics

A total of 1331 patients were enrolled, and 978 received letermovir for CMV prophylaxis. The median duration from transplantation to letermovir prophylaxis was 13 days (range, 0−187 days), with a median duration of 65 days (range, 12−282 days) (**Table 1**). Among these patients, 724 patients discontinued letermovir within 100 days after allo-HSCT, and 254 patients continued letermovir prophylaxis beyond day 100 posttransplant, with a median time of 119 days (range, 101–389 days) after allo-HSCT.

**Table 1:** Patient characteristics before and after PSM adjustment in the total cohort.

Variable	Total cohort		*P*	PSM adjusted		*P*
	Letermovir (*n*=978)	Control (*n*=353)		Letermovir (*n*=216)	Control (*n*=216)	
Age at allo-HSCT			0.140			0.464
16 to 60 years	802 (82.0)	280 (79.3)		164 (76.0)	156 (72.2)	
<16 years	104 (10.6)	51 (14.5)		37 (17.1)	38 (17.6)	
>60 years	72 (7.4)	22 (6.2)		15 (6.9)	22 (10.2)	
Sex, *n* (%)			0.048			0.441
Male	553 (56.5)	178 (50.4)		115 (53.2)	107 (49.5)	
Female	425 (43.5)	175 (49.6)		101 (46.8)	109 (50.5)	
Underlying disease, *n* (%)			0.250			0.771
Acute myeloid leukemia	583 (59.6)	198 (56.1)		120 (55.6)	123 (56.9)	
Acute lymphoblastic leukemia	395 (40.4)	155 (43.9)		96 (44.4)	93 (43.1)	
Donor type, *n* (%)			<0.001			1.000
Matched sibling donor	46 (4.7)	183 (51.8)		46 (21.3)	46 (21.3)	
Haploidentical related donor	863 (88.2)	161 (45.6)		161 (74.5)	161 (74.5)	
Unrelated donor	69 (7.1)	9 (2.6)		9 (4.2)	9 (4.2)	
Donor-recipient sex matched, *n* (%)			0.095			0.786
Others	812 (83.0)	279 (79.0)		185 (85.7)	183 (84.7)	
Female to male	166 (17.0)	74 (21.0)		31 (14.3)	33 (15.3)	
ABO-matched graft, *n* (%)			0.108			1.000
Matched	511 (52.3)	202 (57.2)		114 (52.8)	114 (52.8)	
Mismatched	467 (47.7)	151 (42.8)		102 (47.2)	102 (47.2)	
Conditioning regimen			0.804			0.686
Chemotherapy-based	926 (94.7)	333 (94.3)		202 (93.5)	204 (94.4)	
TBI-based	52 (5.30)	20 (5.7)		14 (6.5)	12 (5.6)	
GVHD prophylaxis			<0.001			0.764
Without ATG	29 (3.0)	121 (34.3)		24 (11.1)	26 (12.0)	
With ATG	949 (97.0)	232 (65.7)		192 (88.9)	190 (88.0)	
CMV serostatus before allo-HSCT			0.131			0.134
Others	925 (94.6)	341 (96.6)		201 (93.1)	208 (96.3)	
Donor-/recipient+	53 (5.4)	12 (3.4)		15 (6.9)	8 (3.7)	
EBV serostatus before allo-HSCT			0.484			1.000
Others	952 (97.3)	346 (98.0)		212 (98.2)	211 (97.7)	
Donor-/recipient+	26 (2.7)	7 (2.0)		4 (1.8)	5 (2.3)	

Allo-HSCT, allogeneic hematopoietic stem cell transplantation; TBI, total body irradiation; GVHD, graft-versus-host disease; ATG, antithymocyte globulin; CMV, cytomegalovirus; EBV, Epstein–Barr virus.

A total of 510 (38.3%) and 73 (5.5%) patients experienced total and grade III to IV aGVHD, respectively, and 443 (33.3%) and 55 (4.1%) patients experienced total and moderate to severe cGVHD, respectively. A total of 142 (10.7%) patients experienced relapse, and 129 (9.7%) patients died of NRM (**Supplementary Table 1**). The median follow-up after allo-HSCT was 510 days (range, 23−1105 days).

### 3.2 CMV infection within 100 days after allo-HSCT

#### 3.2.1 In patients receiving letermovir prophylaxis

Among the patients who received letermovir prophylaxis, 69 (7.1%) had a cs-CMV infection within 100 days, and 52 (5.3%) had a cs-CMV infection during letermovir prophylaxis. The median time from allo-HSCT to cs-CMV infection was 30 days (range 12−99 days). In particular, 2 (0.2%) patients had CMV pneumonia within 100 days (during letermovir prophylaxis). The 100-day cumulative incidence of cs-CMV infection and CMV disease after allo-HSCT was 7.1% (95% CI, 5.5−8.7%) and 0.1% (95% CI, 0−0.3%), respectively, for patients receiving letermovir prophylaxis. The results of the subgroup analysis for the occurrence of cs-CMV infection and CMV disease at 100 days after allo-HSCT are presented in **Table 2**. The 100-day cumulative incidence of cs-CMV was greater in patients who received a TBI-based conditioning regimen (*P* = 0.002) and in children (< 16 years) (*P* = 0.036). Multivariate analysis revealed that no variable was associated with cs-CMV infection or CMV disease at 100 days after allo-HSCT and that HID HSCT with ATG for GVHD prophylaxis did not increase the risk of cs-CMV infection (**Supplementary Table 2**).

**Table 2:** Subgroup analysis for the occurrence of cs-CMV infection and CMV disease at 100 days in patients receiving letermovir prophylaxis.

Subgroup	Cumulative incidence (95% CI)	HR (95% CI)	*P*
cs-CMV infection at 100 days			
Age at allo-HSCT			
16 to 60 years	6.1% (4.5–7.8%)	1.00	
<16 years	11.5% (5.4–17.7%)	1.99 (1.05-3.77)	0.035
>60 years	11.1% (3.8–18.4%)	1.89 (0.89-4.00)	0.098
Sex			
Male	8.3% (6.0–10.7%)	1.00	
Female	5.4% (3.3–7.6%)	0.64 (0.39-1.05)	0.078
Underlying disease			
Acute myeloid leukemia	6.6% (4.5–8.6%)	1.00	
Acute lymphoblastic leukemia	7.9% (5.2–10.5%)	1.23 (0.76-1.97)	0.401
Donor type			
Matched sibling donor	8.9% (0.5–17.3%)	1.00	
Haploidentical related donor	7.2% (5.5–8.9%)	0.83 (0.31-2.23)	0.705
Unrelated donor	4.4% (0.0–9.4%)	0.49 (0.11-2.12)	0.338
Donor-recipient sex matched			
Others	6.8% (5.1–8.5%)	1.00	
Female to male	8.4% (4.2–12.7%)	1.27 (0.70-2.29)	0.426
ABO-matched graft			
Matched	6.9% (4.7–9.1%)	1.00	
Mismatched	7.3% (5.0–9.7%)	1.06 (0.66-1.70)	0.796
Conditioning regimen			
Chemotherapy-based	6.5% (4.9–8.1%)	1.00	
TBI-based	17.4% (6.9–27.8%)	2.96 (1.44-6.07)	0.003
GVHD prophylaxis			
Without ATG	7.1% (0.0–16.9%)	1.00	
With ATG	7.1% (5.5–8.7%)	1.03 (0.26-4.12)	0.963
CMV serostatus before allo-HSCT			
Others	7.3% (5.6–9.0%)	1.00	
Donor-/recipient+	3.8% (0.0–9.0%)	0.51 (0.13-2.06)	0.343
EBV serostatus before allo-HSCT			
Others	7.2% (5.5–8.8%)	1.00	
Donor-/recipient+	3.8% (0.0–11.4%)	0.54 (0.07-3.94)	0.540
Acute GVHD			
Others	8.3% (6.4–10.2%)	1.00	
Grade II to IV	1.2% (0.0–2.9%)	0.32 (0.08-1.34)	0.119

Allo-HSCT, allogeneic hematopoietic stem cell transplantation; TBI, total body irradiation; GVHD, graft-versus-host disease; ATG, antithymocyte globulin; CMV, cytomegalovirus; EBV, Epstein–Barr virus.

#### 3.2.2 Comparison between patients with and without letermovir prophylaxis

**Figure 2:**
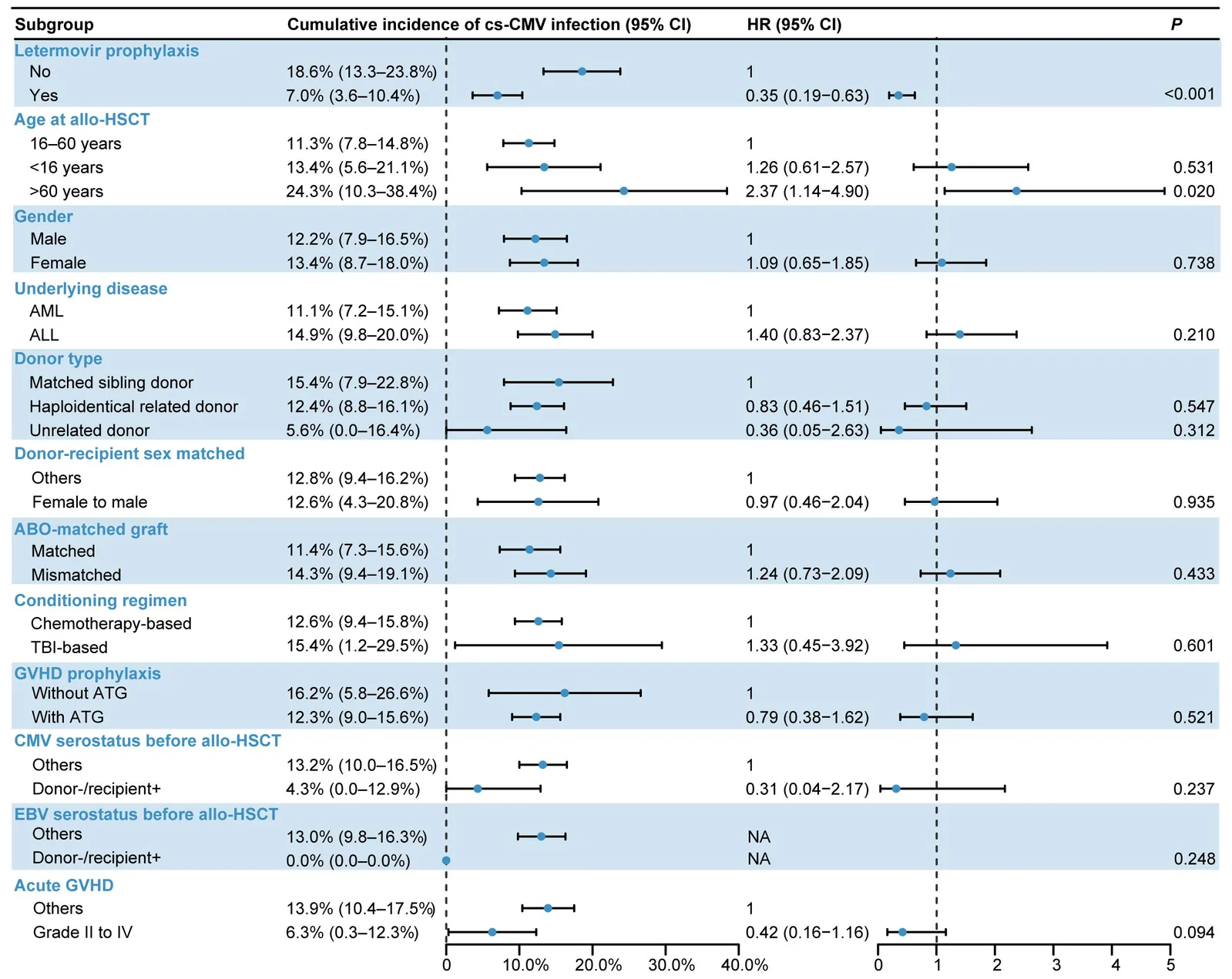
Comparison of cs-CMV infection at 100 days after allo-HSCT after adjustment for PSM. Allo-HSCT, allogeneic hematopoietic stem cell transplantation; AML, acute myeloid leukemia; ALL, acute lymphoblastic leukemia; ATG, antithymocyte globulin; TBI, total body irradiation; CMV, cytomegalovirus; EBV, Epstein–Barr virus; GVHD, graft-versus-host disease; HR, hazard ratio; 95% CI, 95% confidence interval.

After PSM adjustment, for patients with and without letermovir prophylaxis, the 100-day cumulative incidence of cs-CMV infection was 7.0% (95% CI, 3.6−10.4%) and 18.6% (95% CI, 13.3−23.8%) (*P* < 0.001), respectively (**Figure 2**). No patients exhibited CMV disease within 100 days after PSM adjustment.

### 3.3 CMV infection between 100 and 200 days after allo-HSCT

#### 3.3.1 In patients receiving letermovir prophylaxis

Among patients who received letermovir prophylaxis, 98 (10.0%) had a cs-CMV infection between 100 and 200 days, with a median time from allo-HSCT to a cs-CMV infection of 138 days (range 103−197 days). Twelve (1.2%) patients had CMV disease between 100 and 200 days (pneumonia: 9; encephalitis: 1; skin: 1; retinitis: 1), with a median time from allo-HSCT to CMV disease of 156 days (range 121–184 days). In particular, 10 and 2 of them had previously experienced cs-CMV infection and CMV disease, respectively, within 100 days after allo-HSCT.

The results of the subgroup analysis for the occurrence of cs-CMV infection and CMV disease between 100 and 200 days after allo-HSCT are shown in **Supplementary Table 3**. The cumulative incidence of cs-CMV infection between 100 and 200 days after allo-HSCT was greater in elderly patients (> 60 years) (*P* = 0.019) and patients with donor-/recipient+ CMV serostatus (*P* < 0.001). The cumulative incidence of CMV disease between 100 and 200 days after allo-HSCT was greater in patients with donor-/recipient+ CMV serostatus (*P* < 0.001). Particularly, for those who did not have CMV infection within 100 days after allo-HSCT, the cumulative incidences of cs-CMV and CMV disease between 100 and 200 days after allo-HSCT were also higher in elderly patients (> 60 years) and patients with donor-/recipient+ CMV serostatus (**Supplementary Table 4**).

The cumulative incidences of cs-CMV infection and CMV disease between 100 and 200 days after allo-HSCT were 12.5% (95% CI, 10.3–14.7%) and 1.6% (95% CI, 0.8–2.4%), respectively, for patients who received letermovir prophylaxis. Particularly, for those who did not have CMV infection within 100 days after allo-HSCT, the cumulative incidence of cs-CMV infection and CMV disease between 100 and 200 days after allo-HSCT was 11.7% (95% CI, 9.5−13.9%) and 1.4% (95% CI, 0.6−2.2%), respectively. Moreover, the cumulative incidences of cs-CMV infection and CMV disease between 100 and 200 days were comparable between patients who received letermovir prophylaxis within 100 days and beyond 100 days after allo-HSCT (**Supplementary Table 5**).

Multivariate analysis revealed that donor-/recipient+ CMV serostatus (HR, 5.00; 95% CI, 3.13−7.97; *P* < 0.001) was associated with a higher risk of cs-CMV infection, and patients aged > 60 years (HR, 4.01; 95% CI, 1.14−14.13; *P* = 0.030) were associated with a higher risk of CMV disease between 100 and 200 days after allo-HSCT. A female donor-to-male recipient combination (HR, 0.45; 95% CI, 0.22−0.93; *P* = 0.031) was associated with a lower risk of cs-CMV infection between 100 and 200 days after allo-HSCT (**Supplementary Table 6**). For patients who did not have CMV infection within 100 days after allo-HSCT, a donor-/recipient+ CMV serostatus was associated with higher risks of cs-CMV infection and CMV disease between 100 and 200 days after allo-HSCT (**Supplementary Table 7**). HID HSCT with ATG for GVHD prophylaxis did not increase the risk of late cs-CMV infection between 100 and 200 days after allo-HSCT.

#### 3.3.2 Comparison between patients with and without letermovir prophylaxis

After PSM adjustment, the cumulative incidence of cs-CMV infection and CMV disease between 100 and 200 days was 8.3% (95% CI, 4.1−12.4%) and 17.5% (95% CI, 7.6−27.5%) (*P* = 0.052) and 0% and 1.7% (95% CI, 0−5.1%) (*P* = 0.061), respectively, for those who did not receive letermovir and those who received letermovir beyond 100 days (**Supplementary Table 8**).

Particularly, for those who did not have CMV infection within 100 days, the 200-day cumulative incidence of cs-CMV infection and CMV disease was 7.2% (95% CI, 3.3–11.1%) versus 14.6% (95% CI, 5.1–24.0%) (*P* = 0.106) and 0% versus 1.8% (95% CI, 0−5.3%) (*P* = 0.084), respectively, for those who did not receive letermovir and those who received letermovir beyond 100 days (**Supplementary Table 9**).

### 3.4 Refractory CMV infection after letermovir prophylaxis

Among the 978 patients who received letermovir prophylaxis, 97 (9.9%) had refractory CMV infection at 200 days.

For patients receiving letermovir prophylaxis, no variable was associated with refractory CMV infection at 100 days after allo-HSCT (**Supplementary Tables 10**, **11**).

Donor-/recipient+ CMV serostatus (HR, 12.21; 95% CI, 7.15−20.88; *P* < 0.001) was associated with a higher risk of refractory CMV infection between 100 and 200 days after allo-HSCT. A female donor-to-male recipient combination (HR, 0.27; 95% CI, 0.09−0.83; *P* = 0.023) was associated with a lower risk of refractory CMV infection between 100 and 200 days after allo-HSCT. HID HSCT with ATG for GVHD prophylaxis did not increase the risk of refractory CMV infection after allo-HSCT.

**Figure 3:**
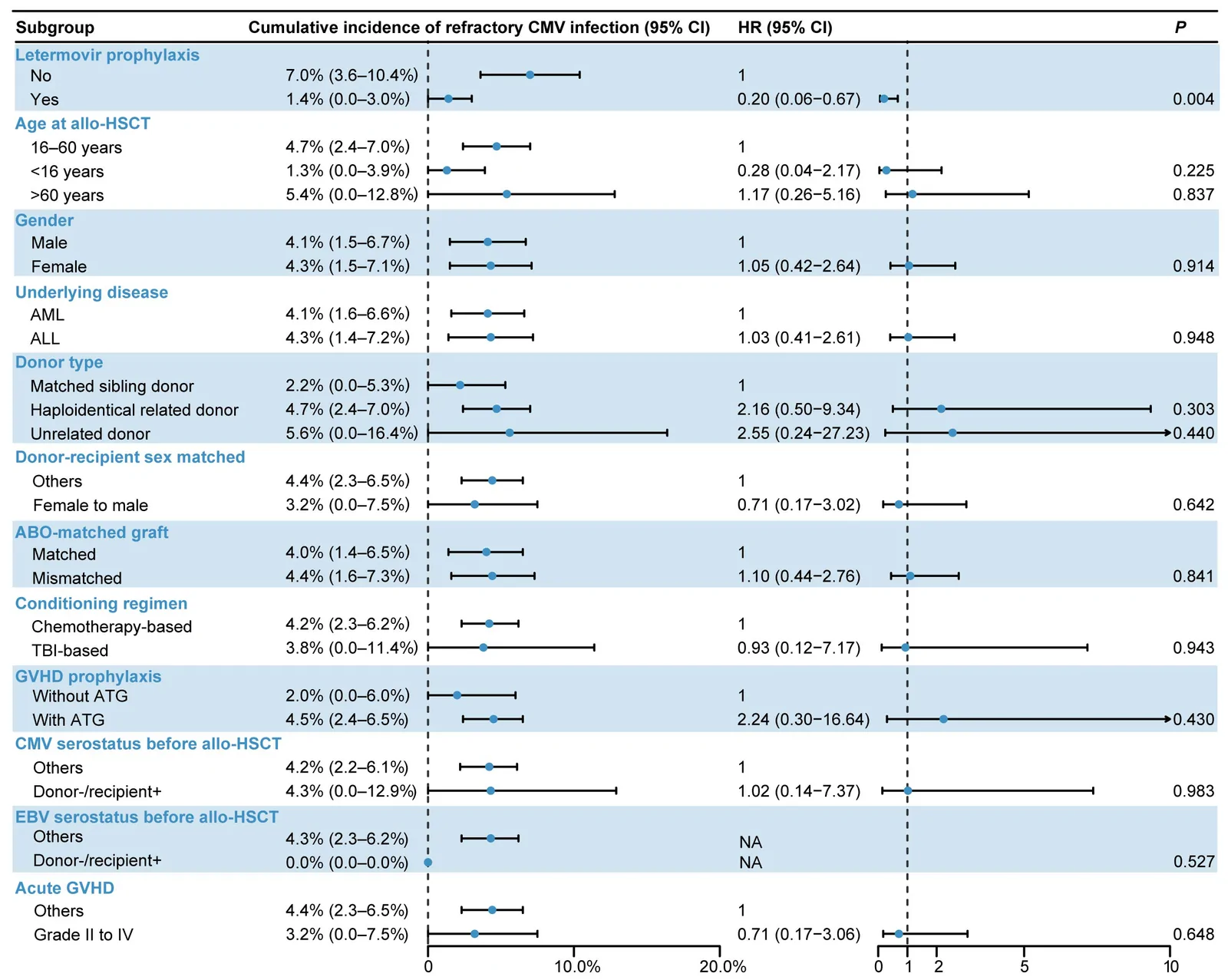
Comparison of refractory CMV infection at 100 days after allo-HSCT after adjustment for PSM. Allo-HSCT, allogeneic hematopoietic stem cell transplantation; AML, acute myeloid leukemia; ALL, acute lymphoblastic leukemia; ATG, antithymocyte globulin; TBI, total body irradiation; CMV, cytomegalovirus; EBV, Epstein–Barr virus; GVHD, graft-versus-host disease; HR, hazard ratio; 95% CI, 95% confidence interval.

The 100-day cumulative incidence of refractory CMV infection after allo-HSCT was 2.0% (95% CI, 1.1−2.9%) for patients who received letermovir prophylaxis. After PSM adjustment, the 100-day cumulative incidence of refractory CMV infection was 7.0% (95% CI, 3.6−10.4%) versus 1.4% (95% CI, 0−3.0%) (*P* = 0.004), respectively, for patients without and with letermovir prophylaxis (**Figure 3**). The cumulative incidence of refractory CMV infection between 100 and 200 days after allo-HSCT was 4.2% (1.4–7.0%), 2.8% (0.1–5.5%), and 10.3% (2.4–18.2%) (*P* = 0.065) for patients who did not receive letermovir, those who received letermovir within 100 days and those who received letermovir beyond 100 days, respectively (**Supplementary Table 12**).

### 3.5 Long-term clinical outcomes after allo-HSCT

The 2-year cumulative incidences of relapse and NRM and the 2-year probabilities of LFS and OS after allo-HSCT for patients who received letermovir prophylaxis were 11.2% (95% CI, 9.1%−13.3%), 12.2% (95% CI, 10.0−14.4%), 76.7% (95% CI, 73.8−79.6%), and 79.2% (95% CI, 76.5−82.0%), respectively.

After PSM adjustment, the 2-year clinical outcomes were comparable among patients who did not receive letermovir, who received letermovir within 100 days and who received letermovir after 100 days (**Supplementary Figure 1**). The results of the multivariate analysis for 2-year clinical outcomes after allo-HSCT are shown in **Supplementary Table 13**.

### 3.6 Letermovir prophylaxis in patients with GVHD

A total of 510 patients experienced aGVHD after allo-HSCT, and 407 received letermovir prophylaxis, with a median duration of 69 days (range 12−282 days). A total of 167 patients experienced grade II to IV aGVHD after allo-HSCT, with a median duration of letermovir prophylaxis of 69 days (range 12–282 days). Among them, 153 patients developed grade II to IV aGVHD during letermovir prophylaxis.

The characteristics of patients with grade II to IV aGVHD are shown in **Supplementary Table 14**. The 100-day cumulative incidence of cs-CMV infection was 14.0% (95% CI, 4.3−23.7%) versus 1.3% (95% CI, 0−3.1%) (*P* < 0.001), respectively, for grade II to IV aGVHD patients without and with letermovir prophylaxis. After PSM adjustment, the 100-day cumulative incidence of cs-CMV infection was 10.7% (95% CI, 0.6−20.8%) versus 1.9% (95% CI, 0−4.6%) (*P* = 0.024), respectively, for grade II to IV aGVHD patients without and with letermovir prophylaxis (**Supplementary Figure 2**).

Sixty-six patients developed grade III to IV aGVHD during letermovir prophylaxis. For these patients, the cumulative incidence of cs-CMV infection was 27.3% (95% CI, 0−55.1%) versus 0% (*P* < 0.001), respectively, for patients without and with letermovir prophylaxis (**Supplementary Figure 2**).

Among the 443 patients who developed cGVHD after allo-HSCT, only 51 received letermovir prophylaxis, with a median prophylaxis duration of 134 days (range, 28–202 days). The cumulative incidence of cs-CMV infection and CMV disease between 100 and 200 days was 18.4% (95% CI, 7.4− 29.4%) and 0%, respectively. Twenty-one patients with moderate or severe cGVHD received letermovir prophylaxis, and the cumulative incidence of cs-CMV infection and CMV disease was 20% (95% CI, 0−59.2%) and 0%, respectively.

### 3.7 Letermovir prophylaxis and EBV infection after allo-HSCT

In the total cohort, 339 patients had cs-EBV infection, and 100 had EBV-PTLD after allo-HSCT. The 2-year cumulative incidence of cs-EBV infection (26.6%, 95% CI, 23.7–29.4% vs. 15.8%, 95% CI, 11.9–19.7%; *P* < 0.001) and EBV-PTLD (9.1%, 95% CI, 7.3–10.9% vs. 4.1%, 95% CI, 2.0–6.2%; *P* = 0.002) among those receiving letermovir prophylaxis was greater than that among those not receiving letermovir prophylaxis (**Supplementary Figure 3**). After PSM adjustment, comparisons of cs-EBV infection and EBV-PTLD between patients with and without letermovir prophylaxis are summarized in **Supplementary Table 15**. Multivariate analysis revealed that among patients who received letermovir prophylaxis, age >60 years (HR, 2.74; 95% CI, 1.77–4.24; *P* < 0.001), a diagnosis of acute lymphoblastic leukemia (HR, 1.48; 95% CI, 1.11–1.98; *P* = 0.008), and HID HSCT (HR, 9.70; 95% CI, 1.18–79.62; *P* = 0.034) were associated with a higher risk of cs-EBV infection. Age >60 years (HR, 5.08; 95% CI, 2.59–9.96; *P* < 0.001) and a TBI-based conditioning regimen (HR, 2.70; 95% CI, 1.18–6.15; *P* = 0.019) were associated with EBV-PTLD after allo-HSCT (**Supplementary Table 16**).

## 4 Discussion

In this large-scale cohort study, we found that the 100-day incidence of cs-CMV infection and refractory CMV infection was only 7% and 1.4%, respectively; these rates were significantly lower than those in patients who did not receive letermovir prophylaxis. We also observed that letermovir decreased the occurrence of cs-CMV infection in patients with aGVHD. However, CMV serostatus donor-/recipient+ still increased the risk of cs-CMV infection and CMV disease, respectively, between 100 and 200 days after allo-HSCT. Thus far, this is the largest cohort study to provide a comprehensive understanding of the use of letermovir for CMV prophylaxis after allo-HSCT in a real-world setting.

HID transplant type is one of the important risk factors for CMV infection, particularly for those with ATG-based GVHD prophylaxis (43, 44). In previous studies, the rate of CMV infection among recipients of HID HSCT with ATG was significantly greater than that among recipients of ISD HSCT (45, 46). Some authors reported that the incidence of CMV infection was comparable between the ISD and HID HSCT groups among patients receiving letermovir prophylaxis, but most of them used PTCy for GVHD prophylaxis, and the sample of those receiving ATG for GVHD prophylaxis was relatively small (47, 48). In our present study, 863 patients undergoing HID HSCT with ATG were included; the 100-day incidence of cs-CMV infection was only 7.2%, and the incidence of refractory CMV infection at 100 days and 100 to 200 days after HID HSCT was only 1.9% and 5.9%, respectively. These findings were all comparable with those observed in ISD HSCT recipients. Letermovir prophylaxis could overcome the poor prognostic effect of the ATG-based HID HSCT regimen on CMV infection in the real world.

Refractory CMV significantly increases the risk of CMV disease and NRM (7, 49). Although several studies have reported that letermovir can reduce the risk of cs-CMV infection (8, 50), whether it can decrease the incidence of refractory CMV infection should be further confirmed. In the study by Sassine et al. (15), a total of 123 patients received letermovir, and the risk of refractory CMV infection decreased; however, the sample of the cohort was relatively small, particularly for the HID recipients (n=20). Yan et al. (51) reported that letermovir could decrease the incidence of refractory CMV infection after umbilical cord blood transplantation. In our large cohort study, we further confirmed that compared with no letermovir prophylaxis, letermovir prophylaxis could significantly decrease the risk of refractory CMV infection after allo-HSCT.

aGVHD patients had a higher risk of CMV infection, and CMV was the most common pathogen after anti-GVHD therapies, such as ruxolitinib and basiliximab (32, 52–54). Thus, whether letermovir could decrease the risk of CMV infection in these patients is worthy of identification, and only some small sample studies have provided positive results for letermovir prophylaxis (55, 56). In the present study, we observed that in patients with aGVHD, receiving letermovir prophylaxis could decrease the risk of cs-CMV infection. Thus, our results confirmed that patients with aGVHD, particularly those with grade III to IV aGVHD, who should receive intensive immunosuppressants could benefit from letermovir prophylaxis.

Several studies have revealed that letermovir can prevent cs-CMV infection in children (57–59), but the sample sizes of these studies were small, and the studies did not compare the efficacy between children and adults. In the present study, we enrolled 104 children (< 16 years old) who received letermovir prophylaxis. Although the incidence of cs-CMV infection was significantly greater in children than in adults (16 to 60 years), the impact of younger age on CMV infection was statistically significant in the univariate analysis but not the multivariate analysis. These findings further confirmed that children can benefit from letermovir prophylaxis.

Several studies have focused on the impact of donor/recipient CMV serostatus before allo-HSCT on the efficacy of letermovir, but the results are controversial. In the study of Johnsrud et al. (24), as many as 50% of the donor-seronegative patients had cs-CMV infection after allo-HSCT. Similarly, Mizuno et al. (13) reported that the cumulative incidence of cs-CMV infection at 200 days was 82.6% in the CMV serostatus donor-/recipient+ group, although they received letermovir prophylaxis, and a CMV serostatus donor-/recipient+ was the only significant factor associated with the development of cs-CMV infection according to multivariate analysis. In contrast, some authors reported that donor/recipient CMV serostatus was not associated with CMV infection after letermovir prophylaxis (60, 61). However, the sample size of the CMV serostatus donor-/recipient+ group was small in the studies mentioned above. In the present study, which included a large CMV serostatus donor-/recipient+ subgroup (*n* = 53), we confirmed that it was still an independent risk factor for 100- to 200-day cs-CMV infection. Thus, methods for preventing CMV infection in these patients should be further identified.

Several studies have reported that CMV-specific cytotoxic T lymphocytes (CMV-CTLs) are important therapeutic methods for CMV infection (62, 63). In addition, Zhao et al. (64) reported that CMV-CTLs could promote the quantitative and functional recovery of CTLs in patients, which suggested that CMV-CTL infusion may help prevent cs-CMV infection (65) even in the era of letermovir prophylaxis for the donor-/recipient+ CMV serostatus. In addition, CMV-specific cell-mediated immunity (CMI) may help direct prolonged letermovir prophylaxis. Wang et al. (66) reported that compared with patients in the fixed-duration group (100-day course of letermovir), patients receiving letermovir on the basis of their CMV-CMI status had a significantly lower rate of late-onset cs-CMV infection (9.7% vs. 24.8%, *P* = 0.019), which can translate to a survival benefit (OS: 89.1% vs. 77.1%, *P* = 0.04; relapse-free survival: 88.4% vs. 76.8%, *P* = 0.01).

We observed that extended letermovir prophylaxis did not further reduce the incidence of late-onset cs-CMV between days 100 and 200 after allo-HSCT, which differs from the findings of Russo et al. (9). These findings suggested that letermovir prophylaxis within 100 days could also prevent cs-CMV infection beyond 100 days after allo-HSCT. However, for the patients who received extended prophylaxis, the actual duration of prophylaxis was relatively short, and the median discontinuation time was only 119 days after allo-HSCT. In the study by Hinman et al. (67), the median duration of letermovir prophylaxis in the extended group was 132 days, and in the real-world study of Masuda et al. (68), the extended group was defined as patients who continued letermovir prophylaxis until day 150 after allo-HSCT. Thus, our results failed to confirm the value of extended letermovir prophylaxis for treating cs-CMV infection between days 100 and 200 after allo-HSCT. In contrast, our study provides a valuable opportunity to identify patients who have a higher risk of late cs-CMV infection in the era of letermovir prophylaxis, such as those whose CMV serostatus is donor-/recipient+ or aged >60 years, and the value of prolonged prophylaxis could be further confirmed in these patients.

Shafat et al. (69) reported that letermovir was not associated with a risk of EBV infection after allo-HSCT, but studies by Kong et al. (26) and Pei et al. (24) showed that letermovir prophylaxis increased the risk of EBV infection. In the present large cohort study, the rates of cs-EBV infection and PTLD were 26.6% and 9.1%, respectively, in patients receiving letermovir prophylaxis, which were higher than those in patients who did not receive letermovir prophylaxis. Huang et al. (25) reported that letermovir may alter the early immune reconstitution trajectory after allo-HCT, particularly in terms of CD8^+^ T cells, which may partially explain the increased risk of cs-EBV infection in these patients. In addition, we reported risk factors for cs-EBV infection and PTLD in patients receiving letermovir prophylaxis, which suggested that these patients may receive further EBV prophylaxis (e.g., EBV CTL infusion (70) or rituximab prophylaxis (71)), which should be further confirmed by prospective studies in the future. The observational nature of this study precludes causal inference. The association may be confounded by unmeasured or residual factors, including the intensity of immunosuppression, donor type, and GVHD, despite adjustment in multivariate analysis.

There were several unsolved problems in this study. First, these patients did not undergo CMI monitoring regularly, and we could not further identify CMI-directed letermovir in the present study. Second, the subgroup findings for grade III–IV aGVHD and cGVHD are limited by small sample sizes and wide confidence intervals, and the protective association of letermovir in these groups needs confirmation in larger prospective studies. In addition, few patients received posttransplant cyclophosphamide (PTCy) for GVHD prevention, and we could not further compare the efficacy of letermovir prophylaxis between the ATG- and PTCy-based regimens. However, this finding is subject to significant treatment-selection bias, as patients receiving extended prophylaxis likely represent a higher-risk cohort. Therefore, the absence of a significant difference in our analysis should not be interpreted as evidence of equivalent efficacy, and the potential benefit of prolonged letermovir in high-risk subgroups requires validation in prospective, randomized studies.

In summary, we comprehensively present the efficacy of letermovir prophylaxis in different subgroups in a large allo-HSCT cohort. For patients with specific risk factors for cs-CMV infection, more aggressive prophylaxis regimens (e.g., CMV-CTL) may be considered. In addition, prolonged letermovir prophylaxis for high-risk patients should be further confirmed in the real world.

## Data Availability

The original contributions presented in the study are included in the article/**Supplementary Material**. Further inquiries can be directed to the corresponding author.
