# Supplementary material for: Letermovir for cytomegalovirus prophylaxis after allogeneic hematopoietic stem cell transplantation in acute leukemia patients: results of the LETreal study
Source: Front Immunol. 2026 Sep 3;17:1923003. doi: 10.3389/fimmu.2026.1923003 (PMC13581788; doi:10.3389/fimmu.2026.1923003)
Supplement: Supplementary file 1 [file SupplementaryFile1.docx]

Supplementary Material

# 1 Supplementary Method

## 1.1 Data collection

Required information was obtained through institutional electronic medical records, including the information on patient demographics, diagnosis, transplant regimen (e.g., conditioning regimens, donor type, donor/recipient CMV serostatus, and GVHD prophylaxis), letermovir prophylaxis, CMV infections (e.g. onset time, clinical manifestation, treatment), Epstein-Barr virus infection, and clinical outcomes (including relapse, mortality, and survival). Two physicians with rich experience in transplantation independently reviewed the data.

## 1.2 Transplantation regimens

Major conditioning regimen consisted of cytarabine, busulfan, cyclophosphamide, and semustine (1-3). Granulocyte colony-stimulating factor primed peripheral blood (PB) harvests were administered to the recipients on the same day of collection. ATG, cyclosporine, mycophenolate mofetil, and short-term methotrexate were administered to prevent GVHD (4). Protocol for acute and chronic GVHD treatment was according to international recommendations (5) and protocols at Peking University Institute of Hematology (PUIH) (6-9).

## 1.3 Infection prophylaxis protocols

Patients were hospitalized in rooms with high-efficiency particulate air (HEPA) filters for 3–4 weeks, that is, from day –10 until the time at which neutrophil engraftment was achieved. All patients received antibiotics for gastrointestinal decontamination during period of conditioning regimen and neutropenia (i.e., fluoroquinolone is orally given to adults and injection liquid of gentamicin is orally given to children). Patients without an invasive fungal disease (IFD) before HSCT received posaconazole from days –10 to +75 to prevent IFD, and those who have an IFD before HID HSCT should receive previously effective antifungal drug from days –9 to +100 to prevent the recurrence of IFD. Patients received trimethoprim-sulfamethoxazole to prevent a *Pneumocystis jirovecii* infection from days –9 to +180.

## 1.4 CMV monitoring and prophylaxis

Quantitative polymerase chain reaction (PCR) analysis for plasma CMV copies was conducted at least weekly until 100 days after allo-HSCT. For the patients who received systemic immunosuppressive therapies beyond 100 days after allo-HSCT, plasma CMV DNA was also monitored at least once every two weeks. Importantly, upon detection of either CMV or EBV reactivation, the monitoring frequency was intensified to twice‑weekly for both viruses until clearance is confirmed, after which the frequency reverted to the baseline schedule according to the post‑transplant time frame.

Ganciclovir (5 mg·kg^-1^) was administered intravenously twice daily for CMV prophylaxis from days –9 to –2 (day 0 being the first day of donor cell infusion), and we used acyclovir (200–400 mg twice daily) after donor stem cell infusion for the prevention of herpes virus which was administered from day +1 until 1 year after HSCT or until the time systemic immunosuppressants were discontinued (10). For patients receiving letermovir for CMV prophylaxis, letermovir was administered at a dose of 480 mg per day (or 240 mg per day in patients taking cyclosporine). For patients receiving letermovir primary prophylaxis who experienced a breakthrough cs-CMV infection within the first 100 days post-transplant, letermovir was resumed after successful viral clearance (confirmed by negative CMV-DNA PCR results) and continued at least until day 100 post-transplant as secondary prophylaxis.

## 1.5 Infection monitoring and prophylaxis other than CMV

Quantitative PCR analysis for plasma EBV copies was conducted at least weekly until 100 days after allo-HSCT. For the patients who received systemic immunosuppressive therapies beyond 100 days after allo-HSCT, plasma EBV DNA was also monitored at least once every two weeks (11-14).

## 1.6 Definition

CMV DNAemia was defined as the detection of CMV DNA in plasma samples. Clinically significant CMV (cs-CMV) infection was defined as CMV disease (15) or CMV DNAemia leading to preemptive treatment on the basis of CMV DNAemia thresholds at our institute (i.e., > 1×103 copies/mL CMV in a single test on plasma) (16). Refractory CMV infection was defined according to the consensus of the Transplant-Associated Virus Infections Forum (15). CMV reactivation was treated in accordance with the Chinese Society of Hematology (17).

EBV DNAemia requiring preemptive treatment was defined as cs-EBV infection, which was based on the thresholds at our institute (i.e., > 1×10^3^ copies/mL EBV in a single test on plasma). EBV-PTLD was defined according to international recommendations (18).

## 1.7 References

1.	Wang Y, Liu QF, Lin R, Yang T, Huang XJ. Optimizing antithymocyte globulin dosing in haploidentical hematopoietic cell transplantation: long-term follow-up of a multicenter, randomized controlled trial. Sci Bull. 2021;66(24):2498-505.

2.	Wang Y, Liu QF, Xu LP, Liu KY, Zhang XH, Ma X, et al. Haploidentical vs identical-sibling transplant for AML in remission: a multicenter, prospective study. Blood. 2015;125(25):3956-62.

3.	Mo X-D, Zhang Y-Y, Zhang X-H, Xu L-P, Wang Y, Yan C-H, et al. The role of collateral related donors in haploidentical hematopoietic stem cell transplantation. Sci Bull. 2018;63(20):1376-82.

4.	Wang Y, Wu DP, Liu QF, Xu LP, Liu KY, Zhang XH, et al. Low-dose post-transplant cyclophosphamide and anti-thymocyte globulin as an effective strategy for GVHD prevention in haploidentical patients. J Hematol Oncol. 2019;12(1):88.

5.	Penack O, Marchetti M, Aljurf M, Arat M, Bonifazi F, Duarte RF, et al. Prophylaxis and management of graft-versus-host disease after stem-cell transplantation for haematological malignancies: updated consensus recommendations of the European Society for Blood and Marrow Transplantation. Lancet Haematol. 2024;11(2):e147-e59.

6.	Cao LQ, Huo WX, Jiang EL, Fu YW, Xu XJ, Lei PC, et al. Xenopax for the treatment of steroid-refractory acute graft-versus-host disease: the RELAX study. Mil Med Res. 2025;12(1):63.

7.	Mo XD, Hong SD, Zhao YL, Jiang EL, Chen J, Xu Y, et al. Basiliximab for steroid-refractory acute graft-versus-host disease: A real-world analysis. Am J Hematol. 2022;97(4):458-69.

8.	Zhao JY, Liu SN, Xu LP, Zhang XH, Wang Y, Chen YH, et al. Ruxolitinib is an effective salvage treatment for multidrug-resistant graft-versus-host disease after haploidentical allogeneic hematopoietic stem cell transplantation without posttransplant cyclophosphamide. Ann Hematol. 2021;100(1):169-80.

9.	Shen MZ, Liu XX, Qiu ZY, Xu LP, Zhang XH, Wang Y, et al. Efficacy and safety of mesenchymal stem cells treatment for multidrug-resistant graft-versus-host disease after haploidentical allogeneic hematopoietic stem cell transplantation. Therapeutic advances in hematology. 2022;13:20406207211072838.

10.	Zaia JA. Cytomegalovirus Infection. In: Stephen J. Forman RSN, Joseph H. Antin and Frederick R. Appelbaum., editor. Thomas’ Hematopoietic Cell Transplantation: Stem Cell Transplantation. Complications of Hematopoietic Cell Transplantation. 2. Fifth ed. UK: John Wiley & Sons, Ltd.; 2016. p. 1069-79.

11.	Hu L, Wang Q, Zhang X, Xu L, Wang Y, Yan C, et al. Positive stool culture could predict the clinical outcomes of haploidentical hematopoietic stem cell transplantation. Front Med. 2019;13(4):492-503.

12.	Mo XD, Zhang XH, Xu LP, Wang Y, Yan CH, Chen H, et al. Late-onset severe pneumonia after allogeneic hematopoietic stem cell transplantation: prognostic factors and treatments. Transpl Infect Dis. 2016;18(4):492-503.

13.	Tomblyn M, Chiller T, Einsele H, Gress R, Sepkowitz K, Storek J, et al. Guidelines for preventing infectious complications among hematopoietic cell transplantation recipients: a global perspective. Biol Blood Marrow Transplant. 2009;15(10):1143-238.

14.	Fan S, Hong HY, Dong XY, Xu LP, Zhang XH, Wang Y, et al. Machine learning algorithm as a prognostic tool for Epstein-Barr virus reactivation after haploidentical hematopoietic stem cell transplantation. Blood Sci. 2023;5(1):51-9.

15.	Ljungman P, Chemaly RF, Khawaya F, Alain S, Avery R, Badshah C, et al. Consensus Definitions of Cytomegalovirus (CMV) Infection and Disease in Transplant Patients Including Resistant and Refractory CMV for Use in Clinical Trials: 2024 Update From the Transplant Associated Virus Infections Forum. Clinical infectious diseases : an official publication of the Infectious Diseases Society of America. 2024;79(3):787-94.

16.	Marty FM, Ljungman P, Chemaly RF, Maertens J, Dadwal SS, Duarte RF, et al. Letermovir Prophylaxis for Cytomegalovirus in Hematopoietic-Cell Transplantation. N Engl J Med. 2017;377(25):2433-44.

17.	[The Chinese consensus on the management of cytomegalovirus infection in allogeneic hematopoietic stem cell transplantation patients (2022)]. Zhonghua xue ye xue za zhi = Zhonghua xueyexue zazhi. 2022;43(8):617-23.

18.	Styczynski J, van der Velden W, Fox CP, Engelhard D, de la Camara R, Cordonnier C, et al. Management of Epstein-Barr Virus infections and post-transplant lymphoproliferative disorders in patients after allogeneic hematopoietic stem cell transplantation: Sixth European Conference on Infections in Leukemia (ECIL-6) guidelines. Haematologica. 2016;101(7):803-11.

# 2 Supplementary Figures and Tables

## 2.1 Supplementary Figures

### 2.1.1 Supplementary Figure 1. Comparison of clinical outcomes after PSM adjustment.

**Supplementary Figure 1.** **Comparison of clinical outcomes after PSM adjustment.** Two-year clinical outcomes after allo-HSCT after PSM adjustment. (A) Relapse; (B) NRM; (C) LFS; (D) OS. Allo-HSCT, allogeneic hematopoietic stem cell transplantation; PSM, propensity score matching; NRM, non-relapse mortality; OS, overall survival; LFS, leukemia-free survival.

### 2.1.2 Supplementary Figure 2. The 100-day cumulative incidence of cs-CMV infection for aGVHD patients after PSM adjustment.

**Supplementary Figure 2.** **The 100-day cumulative incidence of cs-CMV infection for aGVHD patients after PSM adjustment.** (A) For patients with grade II to IV aGVHD; (B) for patients with grade III to IV aGVHD. aGVHD, acute graft-versus-host disease; cs-CMV, clinically significant cytomegalovirus; PSM, propensity score matching.

### 2.1.3 Supplementary Figure 3. The 2-year cumulative incidence of cs-EBV infection and EBV-PTLD.

**Supplementary Figure 3.** **The 2-year cumulative incidence of cs-EBV infection and EBV-PTLD.** (A) cs-EBV infection; (B) EBV-PTLD. EBV, Epstein-Barr virus; PTLD, EBV-associated post-transplant lymphoproliferative disorders.

## 2.2 Supplementary Tables

### 2.2.1 Supplementary Table 1. Causes of NRM.

| **Cause of NRM** | **N (%)** |
| --- | --- |
| Total | 129 (100.0) |
| Infection | 71 (55.0) |
| cs-CMV infection | 4 (3.1) |
| cs-EBV infection | 12 (9.3) |
| Other viral infection | 5 (3.8) |
| Fungal infection | 4 (3.1) |
| Severe pneumonia of unknown etiology | 46 (35.6) |
| Cerebrovascular disease | 8 (6.2) |
| Graft-versus-host disease | 16 (12.4) |
| Transplant-associated thrombotic microangiopathy | 5 (3.8) |
| Multiple organ failure | 10 (7.7) |
| Others | 19 (14.7) |

Abbreviations: NRM, non-relapse mortality; cs-CMV, clinically significant cytomegalovirus; cs-EBV, clinically significant Epstein-Barr virus.

### 2.2.2 Supplementary Table 2. Multivariate analysis of risk factors for CMV infection after allo-HSCT in patients receiving letermovir prophylaxis

| **Variable** | **HR (95% CI)** | ***P*** |
| --- | --- | --- |
| cs-CMV infection at 100 days |  |  |
| Age at allo-HSCT |  |  |
| 16 to 60 years | 1.00 |  |
| <16 years | 1.46 (0.68-3.15) | 0.331 |
| >60 years | 1.91 (0.90-4.06) | 0.094 |
| Gender |  |  |
| Male | 1.00 |  |
| Female | 0.69 (0.41-1.15) | 0.157 |
| Conditioning regimen |  |  |
| Chemotherapy-based | 1.00 |  |
| TBI-based | 2.39 (0.99-5.79) | 0.054 |

Abbreviations: cs-CMV, clinically significant cytomegalovirus; TBI, total-body irradiation.

### 2.2.3 Supplementary Table 3. The subgroup analysis for occurrence of cs-CMV infection and CMV disease between 100 and 200 days for patients receiving letermovir prophylaxis.

| **Subgroup** | **Cumulative incidence (95% CI)** | **HR (95% CI)** | ***P*** |
| --- | --- | --- | --- |
| **cs-CMV infection between 100 and 200 days** |  |  |  |
| Age at allo-HSCT |  |  |  |
| 16 to 60 years | 12.3% (9.9–14.7%) | 1.00 |  |
| <16 years | 6.8% (1.5–12.0%) | 0.53 (0.23–1.20) | 0.128 |
| >60 years | 23.5% (12.2–34.8%) | 1.88 (1.08–3.27) | 0.025 |
| Gender |  |  |  |
| Male | 10.8% (8.0–13.6%) | 1.00 |  |
| Female | 14.6% (11.0–18.1%) | 1.39 (0.95–2.03) | 0.090 |
| Underlying disease |  |  |  |
| Acute myeloid leukemia | 12.1% (9.3–15.0%) | 1.00 |  |
| Acute lymphoblastic leukemia | 13.0% (9.4–16.5%) | 1.09 (0.74–1.60) | 0.671 |
| Donor type |  |  |  |
| Matched sibling donor | 2.5% (0.0–7.4%) | 1.00 |  |
| Haploidentical related donor | 12.5% (10.1–14.8%) | 5.21 (0.72–37.98) | 0.103 |
| Unrelated donor | 19.1% (8.8–29.3%) | 8.09 (1.03–63.49) | 0.047 |
| Donor-recipient sex matched |  |  |  |
| Others | 13.6% (11.1–16.2%) | 1.00 |  |
| Female to male | 6.4% (2.4–10.5%) | 0.46 (0.23–0.90) | 0.024 |
| ABO-matched graft |  |  |  |
| Matched | 12.6% (9.5–15.7%) | 1.00 |  |
| Mismatched | 12.3% (9.1–15.5%) | 0.98 (0.67–1.43) | 0.902 |
| Conditioning regimen |  |  |  |
| Chemotherapy-based | 12.6% (10.3–14.9%) | 1.00 |  |
| TBI-based | 9.8% (0.5–19.1%) | 0.74 (0.28–1.98) | 0.550 |
| GVHD prophylaxis |  |  |  |
| Without ATG | 7.7% (0.0–18.1%) | 1.00 |  |
| With ATG | 12.6% (10.3–14.9%) | 1.68 (0.42–6.79) | 0.463 |
| CMV serostatus before allo-HSCT |  |  |  |
| Others | 10.4% (8.3–12.5%) | 1.00 |  |
| Donor-/recipient+ | 44.2% (30.5–57.9%) | 5.10 (3.26–7.97) | <0.001 |
| EBV serostatus before allo-HSCT |  |  |  |
| Others | 12.7% (10.4–14.9%) | 1.00 |  |
| Donor-/recipient+ | 4.5% (0.0–13.5%) | 0.36 (0.05–2.66) | 0.318 |
| Acute GVHD |  |  |  |
| Others | 12.1% (9.7–14.6%) | 1.00 |  |
| Grade II to IV | 13.9% (8.3–19.4%) | 1.17 (0.72–1.90) | 0.517 |
| Chronic GVHD at 200 days |  |  |  |
| No | 15.0% (11.9–18.0%) | 1.00 |  |
| Yes | 8.1% (5.1–11.2%) | 1.36 (0.86–2.16) | 0.191 |
| CMV disease between 100 and 200 days |  |  |  |
| Age at allo-HSCT |  |  |  |
| 16 to 60 years | 1.2% (0.4–2.0%) | 1.00 |  |
| <16 years | 2.0% (0.0–4.9%) | 1.65 (0.36–7.59) | 0.520 |
| >60 years | 5.0% (0.0–10.6%) | 4.11 (1.12–15.17) | 0.034 |
| Gender |  |  |  |
| Male | 1.8% (0.6–2.9%) | 1.00 |  |
| Female | 1.3% (0.2–2.4%) | 0.72 (0.24–2.16) | 0.561 |
| Underlying disease |  |  |  |
| Acute myeloid leukemia | 1.9% (0.7–3.0%) | 1.00 |  |
| Acute lymphoblastic leukemia | 1.1% (0.0–2.2%) | 0.59 (0.19–1.89) | 0.378 |
| Donor type |  |  |  |
| Matched sibling donor | 0.0% (0.0–0.0%) | NA |  |
| Haploidentical related donor | 1.4% (0.6–2.2%) | NA | 0.437 |
| Unrelated donor | 4.9% (0.0–10.4%) | NA | 0.143 |
| Donor-recipient sex matched |  |  |  |
| Others | 1.7% (0.8–2.7%) | 1.00 |  |
| Female to male | 0.7% (0.0–2.0%) | 0.38 (0.05–2.90) | 0.351 |
| ABO-matched graft |  |  |  |
| Matched | 1.1% (0.1–2.0%) | 1.00 |  |
| Mismatched | 2.1% (0.7–3.5%) | 2.00 (0.67–5.97) | 0.214 |
| Conditioning regimen |  |  |  |
| Chemotherapy-based | 1.5% (0.7–2.4%) | 1.00 |  |
| TBI-based | 2.1% (0.0–6.2%) | 1.33 (0.18–10.01) | 0.784 |
| GVHD prophylaxis |  |  |  |
| Without ATG | 0.0% (0.0–0.0%) | NA |  |
| With ATG | 1.6% (0.8–2.4%) | NA | 0.507 |
| CMV serostatus before allo-HSCT |  |  |  |
| Others | 1.2% (0.5–1.9%) | 1.00 |  |
| Donor-/recipient+ | 7.5% (0.4–14.7%) | 6.62 (2.08–21.07) | 0.001 |
| EBV serostatus before allo-HSCT |  |  |  |
| Others | 1.6% (0.8–2.4%) | NA |  |
| Donor-/recipient+ | 0.0% (0.0–0.0%) | NA | 0.549 |
| Acute GVHD |  |  |  |
| Others | 1.5% (0.6–2.3%) | 1.00 |  |
| Grade II to IV | 2.0% (0.0–4.2%) | 1.34 (0.37–4.79) | 0.657 |
| Chronic GVHD at 200 days |  |  |  |
| No | 1.9% (0.8–3.0%) | 1.00 |  |
| Yes | 1.0% (0.0–2.1%) | 0.52 (0.14–1.86) | 0.313 |

Abbreviations: Allo-HSCT, allogeneic hematopoietic stem cell transplantation; TBI, total body irradiation; GVHD, graft-versus-host disease; ATG, antithymocyte globulin; CMV, cytomegalovirus; EBV, Epstein-Barr virus. NA, not applicable.

### 2.2.4 Supplementary Table 4. The subgroup analysis for occurrence of cs-CMV infection and CMV disease between 100 and 200 days for patients receiving letermovir without CMV infection within 100 days.

| **Subgroup** | **Cumulative incidence (95% CI)** | **HR (95% CI)** | ***P*** |
| --- | --- | --- | --- |
| cs-CMV infection between 100 and 200 days |  |  |  |
| Age at allo-HSCT |  |  |  |
| 16 to 60 years | 11.5% (9.1–13.9%) | 1.00 |  |
| <16 years | 5.7% (0.8–10.6%) | 0.48 (0.19–1.17) | 0.107 |
| >60 years | 23.5% (12.2–34.8%) | 2.02 (1.16–3.53) | 0.013 |
| Gender |  |  |  |
| Male | 10.1% (7.4–12.8%) | 1.00 |  |
| Female | 13.7% (10.2–17.2%) | 1.39 (0.94–2.07) | 0.099 |
| Underlying disease |  |  |  |
| Acute myeloid leukemia | 11.7% (8.9–14.5%) | 1.00 |  |
| Acute lymphoblastic leukemia | 11.7% (8.2–15.1%) | 1.01 (0.68–1.52) | 0.956 |
| Donor type |  |  |  |
| Matched sibling donor | 2.5% (0.0–7.4%) | 1.00 |  |
| Haploidentical related donor | 11.7% (9.4–14.0%) | 4.87 (0.67–35.55) | 0.119 |
| Unrelated donor | 17.9% (7.7–28.0%) | 7.63 (0.96–60.57) | 0.054 |
| Donor-recipient sex matched |  |  |  |
| Others | 12.8% (10.4–15.3%) | 1.00 |  |
| Female to male | 5.8% (1.9–9.8%) | 0.44 (0.21–0.90) | 0.025 |
| ABO-matched graft |  |  |  |
| Matched | 11.8% (8.8–14.8%) | 1.00 |  |
| Mismatched | 11.6% (8.4–14.7%) | 0.97 (0.66–1.45) | 0.894 |
| Conditioning regimen |  |  |  |
| Chemotherapy-based | 11.9% (9.7–14.2%) | 1.00 |  |
| TBI-based | 7.5% (0.0–15.9%) | 0.60 (0.19–1.87) | 0.381 |
| GVHD prophylaxis |  |  |  |
| Without ATG | 7.7% (0.0–18.1%) | 1.00 |  |
| With ATG | 11.8% (9.6–14.0%) | 1.57 (0.39–6.35) | 0.526 |
| CMV serostatus before allo-HSCT |  |  |  |
| Others | 9.5% (7.5–11.6%) | 1.00 |  |
| Donor-/recipient+ | 45.1% (31.2–58.9%) | 5.74 (3.64–9.04) | <0.001 |
| EBV serostatus before allo-HSCT |  |  |  |
| Others | 11.9% (9.7–14.1%) | 1.00 |  |
| Donor-/recipient+ | 4.5% (0.0–13.5%) | 0.39 (0.05–2.87) | 0.353 |
| Acute GVHD |  |  |  |
| Others | 11.2% (8.8–13.6%) | 1.00 |  |
| Grade II to IV | 13.9% (8.3–19.4%) | 1.28 (0.79–2.08) | 0.320 |
| Chronic GVHD at 200 days |  |  |  |
| No | 13.8% (10.9–16.7%) | 1.00 |  |
| Yes | 8.1% (5.1–11.2%) | 1.48 (0.93–2.36) | 0.102 |
| CMV disease between 100 and 200 days |  |  |  |
| Age at allo-HSCT |  |  |  |
| 16 to 60 years | 2.3% (0.0–5.5%) | 1.00 |  |
| <16 years | 1.0% (0.3–1.8%) | 2.28 (0.48–10.91) | 0.300 |
| >60 years | 5.4% (0.0–11.5%) | 5.48 (1.42–21.18) | 0.014 |
| Gender |  |  |  |
| Male | 1.7% (0.5–2.9%) | 1.00 |  |
| Female | 1.1% (0.0–2.1%) | 0.63 (0.19–2.09) | 0.450 |
| Underlying disease |  |  |  |
| Acute myeloid leukemia | 1.6% (0.5–2.7%) | 1.00 |  |
| Acute lymphoblastic leukemia | 1.2% (0.0–2.4%) | 0.76 (0.23–2.51) | 0.650 |
| Donor type |  |  |  |
| Matched sibling donor | 0.0% (0.0–0.0%) | NA |  |
| Haploidentical related donor | 1.4% (0.5–2.2%) | NA | 0.459 |
| Unrelated donor | 3.5% (0.0–8.3%) | NA | 0.235 |
| Donor-recipient sex matched |  |  |  |
| Others | 1.6% (0.7–2.5%) | 1.00 |  |
| Female to male | 0.7% (0.0–2.1%) | 0.46 (0.06–3.57) | 0.458 |
| ABO-matched graft |  |  |  |
| Matched | 0.9% (0.0–1.8%) | 1.00 |  |
| Mismatched | 2.0% (0.6–3.4%) | 2.24 (0.67–7.43) | 0.189 |
| Conditioning regimen |  |  |  |
| Chemotherapy-based | 1.4% (0.6–2.2%) | 1.00 |  |
| TBI-based | 2.6% (0.0–7.6%) | 1.82 (0.24–13.86) | 0.565 |
| GVHD prophylaxis |  |  |  |
| Without ATG | 0.0% (0.0–0.0%) | NA |  |
| With ATG | 1.5% (0.7–2.3%) | NA | 0.532 |
| CMV serostatus before allo-HSCT |  |  |  |
| Others | 1.0% (0.3–1.7%) | 1.00 |  |
| Donor-/recipient+ | 7.8% (0.4–15.3%) | 8.06 (2.42–26.80) | 0.001 |
| EBV serostatus before allo-HSCT |  |  |  |
| Others | 1.5% (0.6–2.3%) | NA |  |
| Donor-/recipient+ | 0.0% (0.0–0.0%) | NA | 0.576 |
| Acute GVHD |  |  |  |
| Others | 1.3% (0.5–2.2%) | 1.00 |  |
| Grade II to IV | 2.0% (0.0–4.2%) | 1.52 (0.41–5.60) | 0.533 |
| Chronic GVHD at 200 days |  |  |  |
| No | 1.7% (0.6–2.8%) | 1.00 |  |
| Yes | 1.0% (0.0–2.1%) | 0.57 (0.15–2.11) | 0.399 |

Abbreviations: Allo-HSCT, allogeneic hematopoietic stem cell transplantation; TBI, total body irradiation; GVHD, graft-versus-host disease; ATG, antithymocyte globulin; CMV, cytomegalovirus; EBV, Epstein-Barr virus. NA, not applicable.

### 2.2.5 Supplementary Table 5. Comparison of cs-CMV infection and CMV disease between patients receiving letermovir within and beyond 100 days after allo-HSCT.

| **Outcome** | **Cumulative incidence (95% CI)** |
| --- | --- |
| Among patients receiving letermovir |  |
| cs-CMV infection between 100 and 200 days |  |
| Letermovir discontinuation time after allo-HSCT |  |
| Within 100 days | 11.2% (8.7–13.7%) |
| Beyond 100 days | 15.6% (11.0–20.3%) |
| CMV disease between 100 and 200 days |  |
| Letermovir discontinuation time after allo-HSCT |  |
| Within 100 days | 1.1% (0.3–1.9%) |
| Beyond 100 days | 2.8% (0.8–4.9%) |
| Among patients receiving letermovir without cs-CMV infection at 100 days |  |
| cs-CMV infection between 100 and 200 days |  |
| Letermovir discontinuation time after allo-HSCT |  |
| Within 100 days | 10.4% (8.0–12.8%) |
| Beyond 100 days | 15.0% (10.4–19.6%) |
| CMV disease between 100 and 200 days |  |
| Letermovir discontinuation time after allo-HSCT |  |
| Within 100 days | 1.0% (0.2–1.8%) |
| Beyond 100 days | 2.6% (0.5–4.6%) |

Abbreviations: Allo-HSCT, allogeneic hematopoietic stem cell transplantation; TBI, total body irradiation; GVHD, graft-versus-host disease; ATG, antithymocyte globulin; CMV, cytomegalovirus; EBV, Epstein-Barr virus.

### 2.2.6 Supplementary Table 6. Multivariate analysis of risk factors for cs-CMV infection between 100 and 200 days after allo-HSCT in patients receiving letermovir prophylaxis.

| **Variable** | **HR (95% CI)** | ***P*** |
| --- | --- | --- |
| cs-CMV infection between 100 and 200 days |  |  |
| Age at allo-HSCT |  |  |
| 16 to 60 years | 1.00 |  |
| <16 years | 0.55 (0.24–1.27) | 0.161 |
| >60 years | 1.65 (0.90–3.04) | 0.106 |
| Gender |  |  |
| Male | 1.00 |  |
| Female | 1.09 (0.73–1.62) | 0.685 |
| Donor type |  |  |
| Matched sibling donor | 1.00 |  |
| Haploidentical related donor | 6.09 (0.81–46.12) | 0.080 |
| Unrelated donor | 6.88 (0.84–56.32) | 0.072 |
| Donor-recipient sex matched |  |  |
| Others | 1.00 |  |
| Female to male | 0.45 (0.22–0.93) | 0.031 |
| Letermovir discontinuation time after allo-HSCT |  |  |
| Within 100 days | 1.00 |  |
| Beyond 100 days | 1.48 (0.99–2.20) | 0.054 |
| CMV serostatus before allo-HSCT |  |  |
| Others | 1.00 |  |
| Donor-/recipient+ | 5.00 (3.13–7.97) | <0.001 |
| CMV disease between 100 and 200 days |  |  |
| Age at allo-HSCT |  |  |
| 16 to 60 years | 1.00 |  |
| <16 years | 2.14 (0.45–10.24) | 0.342 |
| >60 years | 4.01 (1.14–14.13) | 0.030 |
| CMV serostatus before allo-HSCT |  |  |
| Others | 1.00 |  |
| Donor-/recipient+ | 6.98 (2.24–21.80) | <0.001 |

Abbreviations: Allo-HSCT, allogeneic hematopoietic stem cell transplantation; TBI, total body irradiation; GVHD, graft-versus-host disease; ATG, antithymocyte globulin; CMV, cytomegalovirus; EBV, Epstein-Barr virus.

### 2.2.7 Supplementary Table 7. Multivariate analysis of risk factors for cs-CMV infection between 100 and 200 days among patients without CMV infection within 100 days after allo-HSCT.

| **Variable** | **HR (95% CI)** | ***P*** |
| --- | --- | --- |
| cs-CMV infection between 100 and 200 days |  |  |
| Age at allo-HSCT |  |  |
| 16 to 60 years | 1.00 |  |
| <16 years | 0.50 (0.20–1.25) | 0.140 |
| >60 years | 1.74 (0.93–3.26) | 0.083 |
| Gender |  |  |
| Male | 1.00 |  |
| Female | 1.08 (0.71–1.65) | 0.718 |
| Donor type |  |  |
| Matched sibling donor | 1.00 |  |
| Haploidentical related donor | 5.78 (0.76–44.17) | 0.091 |
| Unrelated donor | 6.69 (0.80–55.93) | 0.080 |
| Donor-recipient sex matched |  |  |
| Others | 1.00 |  |
| Female to male | 0.43 (0.20–0.92) | 0.030 |
| CMV serostatus before allo-HSCT |  |  |
| Others | 1.00 |  |
| Donor-/recipient+ | 5.60 (3.47–9.01) | <0.001 |
| Letermovir discontinuation time after allo-HSCT |  |  |
| Within 100 days | 1.00 |  |
| Beyond 100 days | 1.45 (0.96–2.19) | 0.079 |
| CMV disease between 100 and 200 days |  |  |
| Age at allo-HSCT |  |  |
| 16 to 60 years | 1.00 |  |
| <16 years | 3.12 (0.62–15.71) | 0.167 |
| >60 years | 5.23 (1.41–19.44) | 0.013 |
| CMV serostatus before allo-HSCT |  |  |
| Others | 1.00 |  |
| Donor-/recipient+ | 8.21 (2.08–32.45) | <0.001 |
| Letermovir discontinuation time after allo-HSCT |  |  |
| Within 100 days | 1.00 |  |
| Beyond 100 days | 2.70 (0.75–9.67) | 0.128 |

Abbreviations: Allo-HSCT, allogeneic hematopoietic stem cell transplantation; TBI, total body irradiation; GVHD, graft-versus-host disease; ATG, antithymocyte globulin; CMV, cytomegalovirus; EBV, Epstein-Barr virus.

### 2.2.8 Supplementary Table 8. Comparison of cs-CMV infection and CMV disease between 100 and 200 days after allo-HSCT after PSM adjustment.

| **Subgroup** | **Cumulative incidence (95% CI)** | **HR (95% CI)** | ***P*** |
| --- | --- | --- | --- |
| cs-CMV infection between 100 and 200 days |  |  |  |
| Letermovir prophylaxis |  |  |  |
| No | 8.3% (4.1–12.4%) | 1.00 |  |
| Letermovir discontinuation beyond 100 days | 17.5% (7.6–27.5%) | 2.23 (0.99–5.01) | 0.052 |
| Age at allo-HSCT |  |  |  |
| 16 to 60 years | 10.7% (7.0–14.4%) | 1.00 |  |
| <16 years | 7.9% (1.2–14.6%) | 0.72 (0.28–1.84) | 0.490 |
| >60 years | 3.7% (0.0–11.0%) | 0.33 (0.05–2.39) | 0.273 |
| Gender |  |  |  |
| Male | 10.3% (5.9–14.7%) | 1.00 |  |
| Female | 9.0% (4.8–13.3%) | 0.88 (0.45–1.70) | 0.700 |
| Underlying disease |  |  |  |
| Acute myeloid leukemia | 8.2% (4.4–11.9%) | 1.00 |  |
| Acute lymphoblastic leukemia | 11.7% (6.6–16.8%) | 1.46 (0.75–2.83) | 0.261 |
| Donor type |  |  |  |
| Matched sibling donor | 3.9% (0.0–8.2%) | 1.00 |  |
| Haploidentical related donor | 10.8% (7.1–14.5%) | 2.84 (0.85–9.51) | 0.090 |
| Unrelated donor | 20.0% (0.0–41.0%) | 5.56 (1.10–28.15) | 0.038 |
| Donor-recipient sex matched |  |  |  |
| Others | 9.7% (6.4–13.0%) | 1.00 |  |
| Female to male | 9.4% (1.5–17.4%) | 0.99 (0.38–2.55) | 0.977 |
| ABO-matched graft |  |  |  |
| Matched | 9.8% (5.6–13.9%) | 1.00 |  |
| Mismatched | 9.6% (5.1–14.1%) | 0.98 (0.50–1.89) | 0.944 |
| Conditioning regimen |  |  |  |
| Chemotherapy-based | 9.7% (6.6–12.9%) | 1.00 |  |
| TBI-based | 9.4% (0.0–22.1%) | 0.92 (0.23–3.69) | 0.905 |
| GVHD prophylaxis |  |  |  |
| Without ATG | 2.4% (0.0–7.0%) | 1.00 |  |
| With ATG | 10.6% (7.3–14.0%) | 4.60 (0.62–34.35) | 0.137 |
| CMV serostatus before allo-HSCT |  |  |  |
| Others | 8.2% (5.3–11.2%) | 1.00 |  |
| Donor-/recipient+ | 31.8% (11.8–51.8%) | 4.15 (1.89–9.11) | <0.001 |
| EBV serostatus before allo-HSCT |  |  |  |
| Others | 9.9% (6.8–13.0%) | NA |  |
| Donor-/recipient+ | 0.0% (0.0–0.0%) | NA | 0.352 |
| aGVHD at 100 days |  |  |  |
| Others | 9.8% (6.4–13.1%) | 1.00 |  |
| Grade II to IV | 9.1% (1.4–16.8%) | 0.92 (0.36–2.34) | 0.857 |
| Chronic GVHD at 200 days |  |  |  |
| No | 11.0% (6.8–15.2%) | 1.00 |  |
| Yes | 7.7% (3.3–12.0%) | 0.67 (0.33–1.37) | 0.272 |
| CMV disease between 100 and 200 days |  |  |  |
| Letermovir prophylaxis |  |  |  |
| No | 0.0% (0.0–0.0%) | NA |  |
| Letermovir discontinuation beyond 100 days | 1.7% (0.0–5.1%) | NA | 0.061 |
| Age at allo-HSCT |  |  |  |
| 16 to 60 years | 0.7% (0.0–1.6%) | 1.00 |  |
| <16 years | 1.4% (0.0–4.2%) | 2.14 (0.20–23.24) | 0.533 |
| >60 years | 3.0% (0.0–9.0%) | 4.59 (0.42–49.71) | 0.210 |
| Gender |  |  |  |
| Male | 1.5% (0.0–3.1%) | 1.00 |  |
| Female | 0.5% (0.0–1.5%) | 0.34 (0.04–3.29) | 0.354 |
| Underlying disease |  |  |  |
| Acute myeloid leukemia | 0.4% (0.0–1.3%) | 1.00 |  |
| Acute lymphoblastic leukemia | 1.7% (0.0–3.7%) | 3.99 (0.42–38.06) | 0.229 |
| Donor type |  |  |  |
| Matched sibling donor | 0.0% (0.0–0.0%) | NA |  |
| Haploidentical related donor | 0.7% (0.0–1.6%) | NA | 0.439 |
| Unrelated donor | 11.5% (0.0–26.9%) | NA | <0.001 |
| Donor-recipient sex matched |  |  |  |
| Others | 1.2% (0.0–2.3%) | NA |  |
| Female to male | 0.0% (0.0–0.0%) | NA | 0.400 |
| ABO-matched graft |  |  |  |
| Matched | 0.9% (0.0–2.2%) | 1.00 |  |
| Mismatched | 1.1% (0.0–2.5%) | 1.13 (0.16–8.00) | 0.906 |
| Conditioning regimen |  |  |  |
| Chemotherapy-based | 0.8% (0.0–1.7%) | 1.00 |  |
| TBI-based | 4.4% (0.0–13.0%) | 5.39 (0.58–50.38) | 0.139 |
| GVHD prophylaxis |  |  |  |
| Without ATG | 0.0% (0.0–0.0%) | NA |  |
| With ATG | 1.1% (0.0–2.2%) | NA | 0.461 |
| CMV serostatus before allo-HSCT |  |  |  |
| Others | 1.0% (0.0–2.1%) | NA |  |
| Donor-/recipient+ | 0.0% (0.0–0.0%) | NA | 0.622 |
| EBV serostatus before allo-HSCT |  |  |  |
| Others | 1.0% (0.0–2.0%) | NA |  |
| Donor-/recipient+ | 0.0% (0.0–0.0%) | NA | 0.776 |
| aGVHD at 100 days |  |  |  |
| Others | 0.9% (0.0–1.9%) | 1.00 |  |
| Grade II to IV | 1.7% (0.0–4.9%) | 1.94 (0.20–18.74) | 0.569 |
| Chronic GVHD at 200 days |  |  |  |
| No | 0.4% (0.0–1.1%) | 1.00 |  |
| Yes | 2.1% (0.0–4.4%) | 5.44 (0.57–51.83) | 0.140 |

Abbreviations: Allo-HSCT, allogeneic hematopoietic stem cell transplantation; TBI, total body irradiation; GVHD, graft-versus-host disease; ATG, antithymocyte globulin; CMV, cytomegalovirus; EBV, Epstein-Barr virus. NA, not applicable.

### 2.2.9 Supplementary Table 9. Comparison of cs-CMV infection and CMV disease between days 100 and 200 after allo-HSCT among patients without cs-CMV infection within the first 100 days after PSM adjustment.

| **Subgroup** | **Cumulative incidence (95% CI)** | **HR (95% CI)** | ***P*** |
| --- | --- | --- | --- |
| cs-CMV infection between 100 and 200 days |  |  |  |
| Letermovir prophylaxis |  |  |  |
| No | 7.2% (3.3–11.1%) | 1.00 |  |
| Letermovir discontinuation beyond 100 days | 14.6% (5.1–24.0%) | 2.08 (0.86–5.06) | 0.106 |
| Age at allo-HSCT |  |  |  |
| 16 to 60 years | 9.7% (6.1-13.3%) | 1.00 |  |
| <16 years | 6.4% (0.3-12.5%) | 0.64 (0.23–1.83) | 0.407 |
| >60 years | 3.7% (0-11.0%) | 0.37 (0.05–2.65) | 0.320 |
| Gender |  |  |  |
| Male | 8.8% (4.7–12.9%) | 1.00 |  |
| Female | 7.5% (3.6–11.4%) | 0.85 (0.41–1.77) | 0.666 |
| Underlying disease |  |  |  |
| Acute myeloid leukemia | 7.3% (3.7–10.8%) | 1.00 |  |
| Acute lymphoblastic leukemia | 9.4% (4.7–14.1%) | 1.31 (0.63–2.71) | 0.464 |
| Donor type |  |  |  |
| Matched sibling donor | 2.6% (0.0–6.3%) | 1.00 |  |
| Haploidentical related donor | 9.4% (5.9–13.0%) | 3.66 (0.85–15.72) | 0.081 |
| Unrelated donor | 13.8% (0.0–32.4%) | 5.56 (0.77–39.88) | 0.088 |
| Donor-recipient sex matched |  |  |  |
| Others | 7.9% (4.9–11.0%) | 1.00 |  |
| Female to male | 9.4% (1.5–17.4%) | 1.22 (0.46–3.22) | 0.687 |
| ABO-matched graft |  |  |  |
| Matched | 7.9% (4.0–11.7%) | 1.00 |  |
| Mismatched | 8.5% (4.2–12.8%) | 1.09 (0.53–2.25) | 0.819 |
| Conditioning regimen |  |  |  |
| Chemotherapy-based | 8.4% (5.4–11.4%) | 1.00 |  |
| TBI-based | 5.0% (0.0–15.0%) | 0.55 (0.08–3.92) | 0.554 |
| GVHD prophylaxis |  |  |  |
| Without ATG | 0.0% (0.0–0.0%) | NA |  |
| With ATG | 9.2% (6.0–12.4%) | NA | 0.040 |
| CMV serostatus before allo-HSCT |  |  |  |
| Others | 6.6% (3.9–9.3%) | 1.00 |  |
| Donor-/recipient+ | 31.8% (11.8–51.8%) | 5.27 (2.33–11.90) | <0.001 |
| EBV serostatus before allo-HSCT |  |  |  |
| Others | 8.4% (5.4–11.3%) | NA |  |
| Donor-/recipient+ | 0.0% (0.0–0.0%) | NA | <0.001 |
| aGVHD at 100 days |  |  |  |
| Others | 8.3% (5.2–11.4%) | 1.00 |  |
| Grade II to IV | 7.4% (0.3–14.4%) | 0.87 (0.31–2.47) | 0.798 |
| Chronic GVHD at 200 days |  |  |  |
| No | 8.5% (4.7–12.3%) | 1.00 |  |
| Yes | 7.7% (3.3–12.0%) | 0.88 (0.42–1.86) | 0.747 |
| CMV disease between 100 and 200 days |  |  |  |
| Letermovir prophylaxis |  |  |  |
| No | 0.0% (0.0–0.0%) | NA |  |
| Letermovir discontinuation beyond 100 days | 1.8% (0.0–5.3%) | NA | 0.084 |
| Age at allo-HSCT |  |  |  |
| 16 to 60 years | 0.7% (0-1.7%) | 1.00 |  |
| <16 years | 1.6% (0-4.8%) | 2.13 (0.20–23.1) | 0.534 |
| >60 years | 3.7% (0-11.0%) | 4.96 (0.46–53.6) | 0.187 |
| Gender |  |  |  |
| Male | 1.6% (0.0–3.5%) | 1.00 |  |
| Female | 0.6% (0.0–1.7%) | 0.35 (0.04–3.35) | 0.363 |
| Underlying disease |  |  |  |
| Acute myeloid leukemia | 0.5% (0.0–1.4%) | 1.00 |  |
| Acute lymphoblastic leukemia | 2.0% (0.0–4.3%) | 4.18 (0.44–39.80) | 0.213 |
| Donor type |  |  |  |
| Matched sibling donor | 0.0% (0.0–0.0%) | NA |  |
| Haploidentical related donor | 0.8% (0.0–1.8%) | NA | 0.446 |
| Unrelated donor | 12.2% (0.0–28.5%) | NA | 0.002 |
| Donor-recipient sex matched |  |  |  |
| Others | 1.3% (0.0–2.6%) | NA |  |
| Female to male | 0.0% (0.0–0.0%) | NA | 0.403 |
| ABO-matched graft |  |  |  |
| Matched | 1.0% (0.0–2.5%) | 1.00 |  |
| Mismatched | 1.2% (0.0–2.9%) | 1.18 (0.17–8.37) | 0.871 |
| Conditioning regimen |  |  |  |
| Chemotherapy-based | 0.9% (0.0–1.9%) | 1.00 |  |
| TBI-based | 5.0% (0.0–15.0%) | 5.43 (0.58–50.56) | 0.137 |
| GVHD prophylaxis |  |  |  |
| Without ATG | 0.0% (0.0–0.0%) | NA |  |
| With ATG | 1.3% (0.0–2.5%) | NA | 0.468 |
| CMV serostatus before allo-HSCT |  |  |  |
| Others | 1.2% (0.0–2.4%) | NA |  |
| Donor-/recipient+ | 0.0% (0.0–0.0%) | NA | 0.606 |
| EBV serostatus before allo-HSCT |  |  |  |
| Others | 1.2% (0.0–2.3%) | NA |  |
| Donor-/recipient+ | 0.0% (0.0–0.0%) | NA | 0.761 |
| aGVHD at 100 days |  |  |  |
| Others | 1.0% (0.0–2.1%) | 1.00 |  |
| Grade II to IV | 1.8% (0.0–5.3%) | 1.80 (0.19–17.48) | 0.611 |
| Chronic GVHD at 200 days |  |  |  |
| No | 0.5% (0.0–1.4%) | 1.00 |  |
| Yes | 2.1% (0.0–4.4%) | 4.47 (0.47–42.54) | 0.193 |

Abbreviations: Allo-HSCT, allogeneic hematopoietic stem cell transplantation; TBI, total body irradiation; GVHD, graft-versus-host disease; ATG, antithymocyte globulin; CMV, cytomegalovirus; EBV, Epstein-Barr virus. NA, not applicable.

### 2.2.10 Supplementary Table 10. The subgroup analysis of risk factors for refractory CMV infection in patients receiving letermovir prophylaxis.

| **Subgroup** | **Cumulative incidence (95% CI)** | **HR (95% CI)** | ***P*** |
| --- | --- | --- | --- |
| Refractory CMV infection at 100 days |  |  |  |
| Age at allo-HSCT |  |  |  |
| 16 to 60 years | 1.9% (0.9–2.8%) | 1.00 |  |
| <16 years | 1.0% (0.0–2.8%) | 0.51 (0.07–3.91) | 0.519 |
| >60 years | 4.2% (0.0–8.8%) | 2.27 (0.65–7.86) | 0.197 |
| Gender |  |  |  |
| Male | 1.8% (0.7–2.9%) | 1.00 |  |
| Female | 2.1% (0.8–3.5%) | 1.18 (0.48–2.90) | 0.720 |
| Underlying disease |  |  |  |
| Acute myeloid leukemia | 2.1% (0.9–3.3%) | 1.00 |  |
| Acute lymphoblastic leukemia | 1.8% (0.5–3.1%) | 0.86 (0.34–2.19) | 0.753 |
| Donor type |  |  |  |
| Matched sibling donor | 2.3% (0.0–6.7%) | 1.00 |  |
| Haploidentical related donor | 1.9% (1.0–2.8%) | 0.84 (0.11–6.30) | 0.862 |
| Unrelated donor | 2.9% (0.0–7.0%) | 1.30 (0.12–14.16) | 0.830 |
| Donor-recipient sex matched |  |  |  |
| Others | 1.7% (0.8–2.6%) | 1.00 |  |
| Female to male | 3.0% (0.4–5.6%) | 1.76 (0.64–4.90) | 0.276 |
| ABO-matched graft |  |  |  |
| Matched | 2.0% (0.8–3.2%) | 1.00 |  |
| Mismatched | 2.0% (0.7–3.2%) | 0.99 (0.40–2.45) | 0.989 |
| Conditioning regimen |  |  |  |
| Chemotherapy-based | 1.7% (0.9–2.6%) | 1.00 |  |
| TBI-based | 5.8% (0.0–12.3%) | 3.47 (1.00–12.00) | 0.050 |
| GVHD prophylaxis |  |  |  |
| Without ATG | 3.6% (0.0–10.6%) | 1.00 |  |
| With ATG | 1.9% (1.0–2.8%) | 0.54 (0.07–4.03) | 0.547 |
| CMV serostatus before allo-HSCT |  |  |  |
| Others | 2.1% (1.1–3.0%) | NA |  |
| Donor-/recipient+ | 0.0% (0.0–0.0%) | NA | 0.290 |
| EBV serostatus before allo-HSCT |  |  |  |
| Others | 1.9% (1.0–2.8%) | 1.00 |  |
| Donor-/recipient+ | 3.8% (0.0–11.4%) | 2.09 (0.27–16.02) | 0.478 |
| Acute GVHD |  |  |  |
| Others | 1.4% (0.3–2.4%) | NA |  |
| Grade II to IV | 0.0% (0.0–0.0%) | NA | 0.370 |
| Refractory CMV infection between 100 and 200 days |  |  |  |
| Age at allo-HSCT |  |  |  |
| 16 to 60 years | 6.3% (4.6–8.1%) | 1.00 |  |
| <16 years | 1.0% (0.0–3.1%) | 0.53 (0.23–1.20) | 0.128 |
| >60 years | 10.3% (2.4–18.1%) | 1.88 (1.08–3.27) | 0.025 |
| Gender |  |  |  |
| Male | 5.2% (3.2–7.1%) | 1.00 |  |
| Female | 7.0% (4.5–9.6%) | 1.37 (0.80–2.35) | 0.248 |
| Underlying disease |  |  |  |
| Acute myeloid leukemia | 5.9% (3.9–7.9%) | 1.00 |  |
| Acute lymphoblastic leukemia | 6.2% (3.7–8.7%) | 1.06 (0.61–1.83) | 0.831 |
| Donor type |  |  |  |
| Matched sibling donor | 0.0% (0.0–0.0%) | NA |  |
| Haploidentical related donor | 5.9% (4.2–7.5%) | NA | 0.059 |
| Unrelated donor | 11.9% (3.6–20.3%) | NA | 0.119 |
| Donor-recipient sex matched |  |  |  |
| Others | 6.8% (5.0–8.6%) | 1.00 |  |
| Female to male | 2.1% (0.0–4.4%) | 0.29 (0.09–0.94) | 0.038 |
| ABO-matched graft |  |  |  |
| Matched | 6.0% (3.9–8.2%) | 1.00 |  |
| Mismatched | 6.0% (3.7–8.2%) | 0.98 (0.57–1.68) | 0.949 |
| Conditioning regimen |  |  |  |
| Chemotherapy-based | 6.1% (4.5–7.7%) | 1.00 |  |
| TBI-based | 4.3% (0.0–10.2%) | 0.70 (0.17–2.89) | 0.624 |
| GVHD prophylaxis |  |  |  |
| Without ATG | 3.7% (0.0–11.0%) | 1.00 |  |
| With ATG | 6.1% (4.5–7.7%) | 1.63 (0.22–12.14) | 0.633 |
| CMV serostatus before allo-HSCT |  |  |  |
| Others | 3.7% (2.4–5.0%) | 1.00 |  |
| Donor-/recipient+ | 41.5% (28.1–55.0%) | 13.38 (7.82–22.90) | <0.001 |
| EBV serostatus before allo-HSCT |  |  |  |
| Others | 6.0% (4.4–7.6%) | 1.00 |  |
| Donor-/recipient+ | 4.5% (0.0–13.5%) | 0.79 (0.11–5.91) | 0.819 |
| Letermovir discontinuation time after allo-HSCT |  |  |  |
| Within 100 days | 5.5% (3.7–7.2%) | 1.00 |  |
| Beyond 100 days | 7.4% (4.1–10.6%) | 1.35 (0.77–2.39) | 0.296 |
| Acute GVHD |  |  |  |
| Others | 5.6% (3.9–7.3%) | 1.00 |  |
| Grade II to IV | 7.8% (3.6–12.1%) | 1.43 (0.75–2.72) | 0.280 |
| Chronic GVHD at 200 days |  |  |  |
| No | 6.4% (4.4–8.4%) | 1.00 |  |
| Yes | 5.2% (2.7–7.7%) | 0.79 (0.44–1.41) | 0.423 |

Abbreviations: Allo-HSCT, allogeneic hematopoietic stem cell transplantation; TBI, total body irradiation; GVHD, graft-versus-host disease; ATG, antithymocyte globulin; CMV, cytomegalovirus; EBV, Epstein-Barr virus.

### 2.2.11 Supplementary Table 11. Multivariate analysis of risk factors for refractory CMV infection in patients receiving letermovir prophylaxis.

| **Variable** | **HR (95% CI)** | ***P*** |
| --- | --- | --- |
| Refractory CMV infection between 100 and 200 days |  |  |
| Age at allo-HSCT |  |  |
| 16 to 60 years | 1.00 |  |
| <16 years | 0.20 (0.03-1.45) | 0.111 |
| >60 years | 1.43 (0.73-2.83) | 0.300 |
| Donor-recipient sex matched |  |  |
| Others | 1.00 |  |
| Female to male | 0.27 (0.09-0.83) | 0.023 |
| CMV serostatus before allo-HSCT |  |  |
| Others | 1.00 |  |
| Donor-/recipient+ | 12.21 (7.15-20.88) | <0.001 |

Abbreviations: Allo-HSCT, allogeneic hematopoietic stem cell transplantation; TBI, total body irradiation; GVHD, graft-versus-host disease; ATG, antithymocyte globulin; CMV, cytomegalovirus; EBV, Epstein-Barr virus.

### 2.2.12 Supplementary Table 12. Comparison of refractory CMV infection and CMV disease between 100 and 200 days after allo-HSCT after PSM adjustment.

| **Subgroup** | **Cumulative incidence (95% CI)** | **HR (95% CI)** | ***P*** |
| --- | --- | --- | --- |
| Refractory CMV infection between 100 and 200 days |  |  |  |
| Letermovir prophylaxis |  |  |  |
| No | 4.2% (1.4–7.0%) | 1.00 |  |
| Letermovir discontinuation within 100 days | 2.8% (0.1–5.5%) | 0.68 (0.20–2.23) | 0.520 |
| Letermovir discontinuation beyond 100 days | 10.3% (2.4–18.2%) | 2.58 (0.90–7.42) | 0.078 |
| Age at allo-HSCT |  |  |  |
| 16 to 60 years | 4.5% (2.1–6.9%) | 1.00 |  |
| <16 years | 1.4% (0.0–4.2%) | 0.31 (0.04–2.34) | 0.255 |
| >60 years | 6.1% (0.0–14.4%) | 1.34 (0.31–5.82) | 0.695 |
| Gender |  |  |  |
| Male | 4.5% (1.6–7.4%) | 1.00 |  |
| Female | 3.7% (1.0–6.3%) | 0.81 (0.30–2.18) | 0.679 |
| Underlying disease |  |  |  |
| Acute myeloid leukemia | 3.2% (0.8–5.5%) | 1.00 |  |
| Acute lymphoblastic leukemia | 5.3% (1.9–8.7%) | 1.72 (0.64–4.62) | 0.278 |
| Donor type |  |  |  |
| Matched sibling donor | 0.0% (0.0–0.0%) | NA |  |
| Haploidentical related donor | 4.2% (1.9–6.5%) | NA | 0.052 |
| Unrelated donor | 24.7% (2.9–46.5%) | NA | <0.001 |
| Donor-recipient sex matched |  |  |  |
| Others | 4.2% (2.0–6.4%) | 1.00 |  |
| Female to male | 3.4% (0.0–8.0%) | 0.81 (0.18–3.53) | 0.774 |
| ABO-matched graft |  |  |  |
| Matched | 5.3% (2.2–8.4%) | 1.00 |  |
| Mismatched | 2.7% (0.4–5.1%) | 0.50 (0.18–1.45) | 0.203 |
| Conditioning regimen |  |  |  |
| Chemotherapy-based | 4.1% (2.1–6.1%) | 1.00 |  |
| TBI-based | 4.4% (0.0–13.0%) | 1.03 (0.14–7.62) | 0.98 |
| GVHD prophylaxis |  |  |  |
| Without ATG | 0.0% (0.0–0.0%) | NA |  |
| With ATG | 4.7% (2.4–6.9%) | NA | 0.127 |
| CMV serostatus before allo-HSCT |  |  |  |
| Others | 2.7% (1.0–4.4%) | 1.00 |  |
| Donor-/recipient+ | 27.3% (8.2–46.4%) | 11.25 (4.16–30.40) | <0.001 |
| EBV serostatus before allo-HSCT |  |  |  |
| Others | 4.2% (2.2–6.2%) | NA |  |
| Donor-/recipient+ | 0.0% (0.0–0.0%) | NA | 0.556 |
| aGVHD at 100 days |  |  |  |
| Others | 3.3% (1.4–5.2%) | 1.00 |  |
| Grade II to IV | 8.6% (1.3–15.9%) | 2.70 (0.94–7.78) | 0.065 |
| Chronic GVHD at 200 days |  |  |  |
| No | 3.2% (1.0–5.5%) | 1.00 |  |
| Yes | 5.5% (1.8–9.2%) | 1.72 (0.65–4.56) | 0.279 |

Abbreviations: Allo-HSCT, allogeneic hematopoietic stem cell transplantation; TBI, total body irradiation; GVHD, graft-versus-host disease; ATG, antithymocyte globulin; CMV, cytomegalovirus; EBV, Epstein-Barr virus. NA, not applicable.

### 2.2.13 Supplementary Table 13. Univariate and multivariate analysis of risk factors for 2-year clinical outcomes.

| **Variable** | **Univariate analysis** | | **Multivariate analysis** | |
| --- | --- | --- | --- | --- |
|  | **HR (95% CI)** | ***P*** | **HR (95% CI)** | ***P*** |
| Relapse |  |  |  |  |
| Letermovir prophylaxis |  |  |  |  |
| No | 1.00 |  |  |  |
| Letermovir discontinuation within 100 days | 0.76 (0.39–1.47) | 0.412 |  |  |
| Letermovir discontinuation beyond 100 days | 0.57 (0.20–1.63) | 0.294 |  |  |
| Age at allo-HSCT |  |  |  |  |
| 16 to 60 years | 1.00 |  |  |  |
| <16 years | 1.02 (0.45–2.32) | 0.961 |  |  |
| >60 years | 2.06 (0.86–4.96) | 0.106 |  |  |
| Gender |  |  |  |  |
| Male | 1.00 |  |  |  |
| Female | 0.73 (0.40–1.34) | 0.316 |  |  |
| Underlying disease |  |  |  |  |
| Acute myeloid leukemia | 1.00 |  |  |  |
| Acute lymphoblastic leukemia | 0.78 (0.42–1.45) | 0.439 |  |  |
| Donor type |  |  |  |  |
| Matched sibling donor | 1.00 |  |  |  |
| Haploidentical related donor | 0.87 (0.43–1.78) | 0.704 |  |  |
| Unrelated donor | 1.18 (0.26–5.41) | 0.827 |  |  |
| Donor-recipient sex matched |  |  |  |  |
| Others | 1.00 |  |  |  |
| Female to male | 1.77 (0.87–3.58) | 0.115 |  |  |
| ABO-matched graft |  |  |  |  |
| Matched | 1.00 |  |  |  |
| Mismatched | 1.07 (0.59–1.95) | 0.819 |  |  |
| Conditioning regimen |  |  |  |  |
| Chemotherapy-based | 1.00 |  |  |  |
| TBI-based | 1.77 (0.63–4.96) | 0.277 |  |  |
| GVHD prophylaxis |  |  |  |  |
| Without ATG | 1.00 |  |  |  |
| With ATG | 0.78 (0.33–1.85) | 0.570 |  |  |
| CMV serostatus before allo-HSCT |  |  |  |  |
| Others | NA |  |  |  |
| Donor-/recipient+ | NA | NA |  |  |
| EBV serostatus before allo-HSCT |  |  |  |  |
| Others | 1.00 |  |  |  |
| Donor-/recipient+ | 1.44 (0.20–10.50) | 0.717 |  |  |
| aGVHD at 100 days |  |  |  |  |
| Others | 1.00 |  |  |  |
| Grade II to IV | 1.36 (0.63–2.93) | 0.435 |  |  |
| Chronic GVHD at 200 days |  |  |  |  |
| No | 1.00 |  |  |  |
| Yes | 0.62 (0.31–1.22) | 0.167 |  |  |
| NRM |  |  |  |  |
| Letermovir prophylaxis |  |  |  |  |
| No | 1.00 |  | 1.00 |  |
| Letermovir discontinuation within 100 days | 1.53 (0.81–2.89) | 0.193 | 1.60 (0.85–3.04) | 0.148 |
| Letermovir discontinuation beyond 100 days | 0.79 (0.27–2.33) | 0.670 | 0.97 (0.32–2.88) | 0.953 |
| Age at allo-HSCT |  |  |  |  |
| 16 to 60 years | 1.00 |  | 1.00 |  |
| <16 years | 0.44 (0.13–1.43) | 0.172 | 0.43 (0.13–1.41) | 0.164 |
| >60 years | 3.07 (1.45–6.48) | 0.003 | 3.12 (1.47–6.64) | 0.003 |
| Gender |  |  |  |  |
| Male | 1.00 |  |  |  |
| Female | 1.04 (0.57–1.90) | 0.904 |  |  |
| Underlying disease |  |  |  |  |
| Acute myeloid leukemia | 1.00 |  |  |  |
| Acute lymphoblastic leukemia | 0.81 (0.43–1.50) | 0.499 |  |  |
| Donor type |  |  |  |  |
| Matched sibling donor | 1.00 |  |  |  |
| Haploidentical related donor | 1.38 (0.61–3.12) | 0.434 |  |  |
| Unrelated donor | 0.82 (0.10–6.66) | 0.852 |  |  |
| Donor-recipient sex matched |  |  |  |  |
| Others | 1.00 |  |  |  |
| Female to male | 1.14 (0.50–2.56) | 0.758 |  |  |
| ABO-matched graft |  |  |  |  |
| Matched | 1.00 |  |  |  |
| Mismatched | 0.70 (0.37–1.30) | 0.258 |  |  |
| Conditioning regimen |  |  |  |  |
| Chemotherapy-based | 1.00 |  |  |  |
| TBI-based | 1.34 (0.41–4.34) | 0.624 |  |  |
| GVHD prophylaxis |  |  |  |  |
| Without ATG | 1.00 |  |  |  |
| With ATG | 0.95 (0.37–2.43) | 0.922 |  |  |
| CMV serostatus before allo-HSCT |  |  |  |  |
| Others | 1.00 |  |  |  |
| Donor-/recipient+ | 0.86 (0.21–3.55) | 0.832 |  |  |
| EBV serostatus before allo-HSCT |  |  |  |  |
| Others | 1.00 |  | 1.00 |  |
| Donor-/recipient+ | 4.48 (1.38–14.55) | 0.012 | 4.52 (1.38–14.82) | 0.013 |
| aGVHD at 100 days |  |  |  |  |
| Others | 1.00 |  |  |  |
| Grade II to IV | 0.78 (0.31–2.00) | 0.610 |  |  |
| Chronic GVHD at 200 days |  |  |  |  |
| No | 1.00 |  |  |  |
| Yes | 0.80 (0.42–1.54) | 0.506 |  |  |
| LFS |  |  |  |  |
| Letermovir prophylaxis |  |  |  |  |
| No | 1.00 |  | 1.00 |  |
| Letermovir discontinuation within 100 days | 1.08 (0.68–1.70) | 0.750 | 1.11 (0.70–1.75) | 0.653 |
| Letermovir discontinuation beyond 100 days | 0.66 (0.31–1.40) | 0.277 | 0.75 (0.35–1.61) | 0.468 |
| Age at allo-HSCT |  |  |  |  |
| 16 to 60 years | 1.00 |  | 1.00 |  |
| <16 years | 0.73 (0.37–1.43) | 0.356 | 0.72 (0.37–1.41) | 0.343 |
| >60 years | 2.60 (1.48–4.58) | <0.001 | 2.57 (1.45–4.55) | 0.001 |
| Gender |  |  |  |  |
| Male | 1.00 |  |  |  |
| Female | 0.87 (0.57–1.33) | 0.517 |  |  |
| Underlying disease |  |  |  |  |
| Acute myeloid leukemia | 1.00 |  |  |  |
| Acute lymphoblastic leukemia | 0.79 (0.51–1.23) | 0.297 |  |  |
| Donor type |  |  |  |  |
| Matched sibling donor | 1.00 |  |  |  |
| Haploidentical related donor | 1.07 (0.63–1.83) | 0.803 |  |  |
| Unrelated donor | 1.00 (0.29–3.41) | 1.000 |  |  |
| Donor-recipient sex matched |  |  |  |  |
| Others | 1.00 |  |  |  |
| Female to male | 1.46 (0.86–2.49) | 0.162 |  |  |
| ABO-matched graft |  |  |  |  |
| Matched | 1.00 |  |  |  |
| Mismatched | 0.87 (0.57–1.34) | 0.529 |  |  |
| Conditioning regimen |  |  |  |  |
| Chemotherapy-based | 1.00 |  |  |  |
| TBI-based | 1.55 (0.71–3.35) | 0.269 |  |  |
| GVHD prophylaxis |  |  |  |  |
| Without ATG | 1.00 |  |  |  |
| With ATG | 0.85 (0.45–1.59) | 0.603 |  |  |
| CMV serostatus before allo-HSCT |  |  |  |  |
| Others | 1.00 |  |  |  |
| Donor-/recipient+ | 0.40 (0.10–1.62) | 0.199 |  |  |
| EBV serostatus before allo-HSCT |  |  |  |  |
| Others | 1.00 |  | 1.00 |  |
| Donor-/recipient+ | 3.00 (1.10–8.19) | 0.032 | 3.02 (1.10–8.30) | 0.032 |
| aGVHD at 100 days |  |  |  |  |
| Others | 1.00 |  |  |  |
| Grade II to IV | 1.06 (0.59–1.91) | 0.845 |  |  |
| Chronic GVHD at 200 days |  |  |  |  |
| No | 1.00 |  |  |  |
| Yes | 0.70 (0.44–1.12) | 0.140 |  |  |
| OS |  |  |  |  |
| Letermovir prophylaxis |  |  |  |  |
| No | 1.00 |  | 1.00 |  |
| Letermovir discontinuation within 100 days | 1.06 (0.65–1.74) | 0.805 | 1.16 (0.71–1.91) | 0.549 |
| Letermovir discontinuation beyond 100 days | 0.57 (0.24–1.36) | 0.207 | 0.71 (0.30–1.69) | 0.441 |
| Age at allo-HSCT |  |  |  |  |
| 16 to 60 years | 1.00 |  | 1.00 |  |
| <16 years | 0.72 (0.34–1.51) | 0.379 | 0.65 (0.31–1.39) | 0.269 |
| >60 years | 3.20 (1.79–5.71) | <0.001 | 3.05 (1.70–5.49) | <0.001 |
| Gender |  |  |  |  |
| Male | 1.00 |  |  |  |
| Female | 0.93 (0.58–1.47) | 0.751 |  |  |
| Underlying disease |  |  |  |  |
| Acute myeloid leukemia | 1.00 |  |  |  |
| Acute lymphoblastic leukemia | 0.74 (0.46–1.20) | 0.224 |  |  |
| Donor type |  |  |  |  |
| Matched sibling donor | 1.00 |  |  |  |
| Haploidentical related donor | 1.23 (0.67–2.26) | 0.496 |  |  |
| Unrelated donor | 1.36 (0.39–4.78) | 0.631 |  |  |
| Donor-recipient sex matched |  |  |  |  |
| Others | 1.00 |  |  |  |
| Female to male | 1.24 (0.68–2.27) | 0.477 |  |  |
| ABO-matched graft |  |  |  |  |
| Matched | 1.00 |  |  |  |
| Mismatched | 1.02 (0.64–1.62) | 0.940 |  |  |
| Conditioning regimen |  |  |  |  |
| Chemotherapy-based | 1.00 |  |  |  |
| TBI-based | 1.61 (0.70–3.73) | 0.261 |  |  |
| GVHD prophylaxis |  |  |  |  |
| Without ATG | 1.00 |  |  |  |
| With ATG | 1.20 (0.55–2.62) | 0.642 |  |  |
| CMV serostatus before allo-HSCT |  |  |  |  |
| Others | 1.00 |  |  |  |
| Donor-/recipient+ | 0.49 (0.12–1.98) | 0.314 |  |  |
| EBV serostatus before allo-HSCT |  |  |  |  |
| Others | 1.00 |  | 1.00 |  |
| Donor-/recipient+ | 3.53 (1.28–9.68) | 0.014 | 3.90 (1.41–10.81) | 0.009 |
| aGVHD at 100 days |  |  |  |  |
| Others | 1.00 |  |  |  |
| Grade II to IV | 0.94 (0.48–1.84) | 0.859 |  |  |
| Chronic GVHD at 200 days |  |  |  |  |
| No | 1.00 |  | 1.00 |  |
| Yes | 0.64 (0.38–1.08) | 0.092 | 0.62 (0.36–1.05) | 0.078 |

Abbreviations: Allo-HSCT, allogeneic hematopoietic stem cell transplantation; TBI, total body irradiation; GVHD, graft-versus-host disease; ATG, antithymocyte globulin; CMV, cytomegalovirus; EBV, Epstein-Barr virus; PTLD, EBV-associated post-transplant lymphoproliferative disorders; NRM, non-relapse mortality; LFS, leukemia-free survival; OS, overall survival. NA, not applicable.

### 2.2.14 Supplementary Table 14. Characteristics of patients with grade II to IV aGVHD before and after PSM adjustment.

| **Variable** | **Grade II to IV aGVHD** | | ***P*** | **PSM adjusted** | | ***P*** |
| --- | --- | --- | --- | --- | --- | --- |
|  | **Letermovir**  **(n=153)** | **Control**  **(n=65)** |  | **Letermovir**  **(n=146)** | **Control**  **(n=52)** |  |
| Age at allo-HSCT |  |  | 0.093 |  |  | 0.086 |
| 16 to 60 years | 129 (84.31) | 47 (72.31) |  | 122 (83.56) | 36 (69.23) |  |
| <16 years | 17 (11.11) | 11 (16.92) |  | 17 (11.64) | 11 (21.15) |  |
| >60 years | 7 (4.58) | 7 (10.77) |  | 7 (4.79) | 5 (9.62) |  |
| Gender, n (%) |  |  | 0.889 |  |  | 0.745 |
| Male | 91 (59.48) | 38 (58.46) |  | 88 (60.27) | 30 (57.69) |  |
| Female | 62 (40.52) | 27 (41.54) |  | 58 (39.73) | 22 (42.31) |  |
| Underlying disease, n (%) |  |  | 0.935 |  |  | 0.773 |
| Acute myeloid leukemia | 88 (57.52) | 37 (56.92) |  | 82 (56.16) | 28 (53.85) |  |
| Acute lymphoblastic leukemia | 65 (42.48) | 28 (43.08) |  | 64 (43.84) | 24 (46.15) |  |
| Donor type, n (%) |  |  | <0.001 |  |  | 0.223 |
| Matched sibling donor | 5 (3.27) | 18 (27.69) |  | 5 (3.42) | 5 (9.62) |  |
| Haploidentical related donor | 133 (86.93) | 44 (67.69) |  | 129 (88.36) | 44 (84.62) |  |
| Unrelated donor | 15 (9.80) | 3 (4.62) |  | 12 (8.22) | 3 (5.77) |  |
| Donor-recipient sex matched, n (%) |  |  | 0.029 |  |  | 0.375 |
| Others | 130 (84.97) | 47 (72.31) |  | 123 (84.25) | 41 (78.85) |  |
| Female to male | 23 (15.03) | 18 (27.69) |  | 23 (15.75) | 11 (21.15) |  |
| ABO-matched graft, n (%) |  |  | 0.190 |  |  | 0.273 |
| Matched | 77 (50.33) | 39 (60.00) |  | 77 (52.74) | 32 (61.54) |  |
| Mismatched | 76 (49.67) | 26 (40.00) |  | 69 (47.26) | 20 (38.46) |  |
| Conditioning regimen |  |  | 0.176 |  |  | 0.098 |
| Chemotherapy-based | 149 (97.39) | 60 (92.31) |  | 142 (97.26) | 47 (90.38) |  |
| TBI-based | 4 (2.61) | 5 (7.69) |  | 4 (2.74) | 5 (9.62) |  |
| GVHD prophylaxis |  |  | <0.001 |  |  | 0.066 |
| Without ATG | 5 (3.27) | 14 (21.54) |  | 5 (3.42) | 6 (11.54) |  |
| With ATG | 148 (96.73) | 51 (78.46) |  | 141 (96.58) | 46 (88.46) |  |
| CMV serostatus before allo-HSCT |  |  | 0.294 |  |  | 0.406 |
| Others | 144 (94.12) | 64 (98.46) |  | 137 (93.84) | 51 (98.08) |  |
| Donor-/recipient+ | 9 (5.88) | 1 (1.54) |  | 9 (6.16) | 1 (1.92) |  |
| EBV serostatus before allo-HSCT |  |  | 1.000 |  |  | 1.000 |
| Others | 148 (96.73) | 63 (96.92) |  | 141 (96.58) | 50 (96.15) |  |
| Donor-/recipient+ | 5 (3.27) | 2 (3.08) |  | 5 (3.42) | 2 (3.85) |  |

Abbreviations: Allo-HSCT, allogeneic hematopoietic stem cell transplantation; TBI, total body irradiation; GVHD, graft-versus-host disease; ATG, antithymocyte globulin; CMV, cytomegalovirus; EBV, Epstein-Barr virus.

### 2.2.15 Supplementary Table 15. Comparison of 2-year cumulative incidence of cs-EBV infection and EBV-PTLD after PSM adjustment.

| **Outcome** | **Cumulative incidence (95% CI)** | ***P*** |
| --- | --- | --- |
| cs-EBV infection |  |  |
| Letermovir prophylaxis |  |  |
| No | 21.6% (16.1–27.1%) |  |
| Yes | 23.6% (17.7–29.5%) | 0.719 |
| EBV-PTLD |  |  |
| Letermovir prophylaxis |  |  |
| No | 6.1% (2.9–9.3%) |  |
| Yes | 6.3% (3.1–9.5%) | 0.709 |

Abbreviations: EBV, Epstein-Barr virus; PTLD, EBV-associated post-transplant lymphoproliferative disorders.

### 2.2.16 Supplementary Table 16. Univariate and multivariate analysis of risk factors for 2-year cs-EBV infection and EBV-PTLD for patients receiving letermovir prophylaxis.

| **Variable** | **Univariate analysis** | | **Multivariate analysis** | |
| --- | --- | --- | --- | --- |
|  | **HR (95% CI)** | ***P*** | **HR (95% CI)** | ***P*** |
| cs-EBV infection |  |  |  |  |
| Age at allo-HSCT |  |  |  |  |
| 16 to 60 years | 1.00 |  | 1.00 |  |
| <16 years | 1.45 (0.97–2.17) | 0.069 | 1.11 (0.71–1.74) | 0.636 |
| >60 years | 2.66 (1.73–4.09) | <0.001 | 2.74 (1.77–4.24) | <0.001 |
| Gender |  |  |  |  |
| Male | 1.00 |  |  |  |
| Female | 1.03 (0.78–1.36) | 0.817 |  |  |
| Underlying disease |  |  |  |  |
| Acute myeloid leukemia | 1.00 |  | 1.00 |  |
| Acute lymphoblastic leukemia | 1.53 (1.16–2.01) | 0.002 | 1.48 (1.11–1.98) | 0.008 |
| Donor type |  |  |  |  |
| Matched sibling donor | 1.00 |  | 1.00 |  |
| Haploidentical related donor | 10.97 (1.54–78.30) | 0.017 | 9.70 (1.18–79.62) | 0.034 |
| Unrelated donor | 5.82 (0.74–45.93) | 0.095 | 5.43 (0.60–49.26) | 0.132 |
| Donor-recipient sex matched |  |  |  |  |
| Others | 1.00 |  |  |  |
| Female to male | 0.95 (0.65–1.39) | 0.802 |  |  |
| ABO-matched graft |  |  |  |  |
| Matched | 1.00 |  |  |  |
| Mismatched | 0.83 (0.63–1.09) | 0.176 |  |  |
| Conditioning regimen |  |  |  |  |
| Chemotherapy-based | 1.00 |  | 1.00 |  |
| TBI-based | 2.24 (1.39–3.59) | <0.001 | 1.68 (0.97–2.89) | 0.062 |
| GVHD prophylaxis |  |  |  |  |
| Without ATG | 1.00 |  | 1.00 |  |
| With ATG | 3.47 (0.86–13.97) | 0.080 | 1.17 (0.26–5.20) | 0.839 |
| CMV serostatus before allo-HSCT |  |  |  |  |
| Others | 1.00 |  |  |  |
| Donor-/recipient+ | 1.06 (0.61–1.86) | 0.832 |  |  |
| EBV serostatus before allo-HSCT |  |  |  |  |
| Others | 1.00 |  |  |  |
| Donor-/recipient+ | 0.96 (0.43–2.16) | 0.917 |  |  |
| Letermovir discontinuation time after allo-HSCT |  |  |  |  |
| Within 100 days | 1.00 |  | 1.00 |  |
| Beyond 100 days | 1.32 (0.99–1.76) | 0.062 | 1.27 (0.95–1.71) | 0.105 |
| Acute GVHD |  |  |  |  |
| Others | 1.00 |  | 1.00 |  |
| Grade II to IV | 0.70 (0.47–1.04) | 0.075 | 0.73 (0.49–1.08) | 0.116 |
| Chronic GVHD at 200 days |  |  |  |  |
| No | 1.00 |  | 1.00 |  |
| Yes | 0.73 (0.55–0.98) | 0.036 | 0.80 (0.60–1.08) | 0.143 |
| EBV-PTLD |  |  |  |  |
| Age at allo-HSCT |  |  |  |  |
| 16 to 60 years | 1.00 |  | 1.00 |  |
| <16 years | 2.23 (1.20–4.13) | 0.011 | 1.57 (0.77–3.21) | 0.213 |
| >60 years | 4.34 (2.24–8.39) | <0.001 | 5.08 (2.59–9.96) | <0.001 |
| Gender |  |  |  |  |
| Male | 1.00 |  |  |  |
| Female | 0.97 (0.61–1.56) | 0.912 |  |  |
| Underlying disease |  |  |  |  |
| Acute myeloid leukemia | 1.00 |  | 1.00 |  |
| Acute lymphoblastic leukemia | 1.68 (1.05–2.70) | 0.031 | 1.63 (0.97–2.71) | 0.063 |
| Donor type |  |  |  |  |
| Matched sibling donor | NA |  |  |  |
| Haploidentical related donor | NA | NA |  |  |
| Unrelated donor | NA | NA |  |  |
| Donor-recipient sex matched |  |  |  |  |
| Others | 1.00 |  |  |  |
| Female to male | 0.79 (0.39–1.59) | 0.505 |  |  |
| ABO-matched graft |  |  |  |  |
| Matched | 1.00 |  |  |  |
| Mismatched | 0.99 (0.62–1.59) | 0.965 |  |  |
| Conditioning regimen |  |  |  |  |
| Chemotherapy-based | 1.00 |  | 1.00 |  |
| TBI-based | 3.69 (1.89–7.21) | <0.001 | 2.70 (1.18–6.15) | 0.019 |
| GVHD prophylaxis |  |  |  |  |
| Without ATG | NA |  |  |  |
| With ATG | NA | NA |  |  |
| CMV serostatus before allo-HSCT |  |  |  |  |
| Others | 1.00 |  |  |  |
| Donor-/recipient+ | 0.46 (0.11–1.88) | 0.279 |  |  |
| EBV serostatus before allo-HSCT |  |  |  |  |
| Others | 1.00 |  |  |  |
| Donor-/recipient+ | 0.98 (0.24–4.01) | 0.981 |  |  |
| Letermovir discontinuation time after allo-HSCT |  |  |  |  |
| Within 100 days | 1.00 |  | 1.00 |  |
| Beyond 100 days | 1.17 (0.71–1.94) | 0.539 | 1.09 (0.66–1.82) | 0.726 |
| Acute GVHD |  |  |  |  |
| Others | 1.00 |  |  |  |
| Grade II to IV | 0.65 (0.32–1.32) | 0.234 |  |  |
| Chronic GVHD at 200 days |  |  |  |  |
| No | 1.00 |  |  |  |
| Yes | 0.87 (0.53–1.42) | 0.566 |  |  |

Abbreviations: Allo-HSCT, allogeneic hematopoietic stem cell transplantation; TBI, total body irradiation; GVHD, graft-versus-host disease; ATG, antithymocyte globulin; CMV, cytomegalovirus; EBV, Epstein-Barr virus; PTLD, EBV-associated post-transplant lymphoproliferative disorders. NA, not applicable.
